# Pan-cancer prognostic genetic mutations and clinicopathological factors associated with survival outcomes: a systematic review

**DOI:** 10.1038/s41698-022-00269-5

**Published:** 2022-04-20

**Authors:** Jurgita Gammall, Alvina G. Lai

**Affiliations:** 1 Institute of Health Informatics, University College London, London, UK; 2 Cerner Limited, London, UK; 3 Health Data Research UK, London, UK

**Keywords:** Prognostic markers, Cancer epidemiology, Cancer genomics, Tumour biomarkers, Prognosis

## Abstract

Cancer is a leading cause of death, accounting for almost 10 million deaths annually worldwide. Personalised therapies harnessing genetic and clinical information may improve survival outcomes and reduce the side effects of treatments. The aim of this study is to appraise published evidence on clinicopathological factors and genetic mutations (single nucleotide polymorphisms [SNPs]) associated with prognosis across 11 cancer types: lung, colorectal, breast, prostate, melanoma, renal, glioma, bladder, leukaemia, endometrial, ovarian. A systematic literature search of PubMed/MEDLINE and Europe PMC was conducted from database inception to July 1, 2021. 2497 publications from PubMed/MEDLINE and 288 preprints from Europe PMC were included. Subsequent reference and citation search was conducted and a further 39 articles added. 2824 articles were reviewed by title/abstract and 247 articles were selected for systematic review. Majority of the articles were retrospective cohort studies focusing on one cancer type, 8 articles were on pan-cancer level and 6 articles were reviews. Studies analysing clinicopathological factors included 908,567 patients and identified 238 factors, including age, gender, stage, grade, size, site, subtype, invasion, lymph nodes. Genetic studies included 210,802 patients and identified 440 gene mutations associated with cancer survival, including genes *TP53, BRCA1, BRCA2, BRAF, KRAS, BIRC5*. We generated a comprehensive knowledge base of biomarkers that can be used to tailor treatment according to patients’ unique genetic and clinical characteristics. Our pan-cancer investigation uncovers the biomarker landscape and their combined influence that may help guide health practitioners and researchers across the continuum of cancer care from drug development to long-term survivorship.

## Introduction

Cancer is a leading cause of death, accounting for about 19 million new cases and almost 10 million deaths annually worldwide^1^. Increasing burden of cancer incidence and mortality has become one of the key public health targets globally, leading to a surge of research focusing on understanding, prevention and treatment of cancer disease.

Traditionally, initial patient treatment and prognosis are defined by cancer type and stage at diagnosis, using widely accepted cancer staging systems such as Tumour-Node-Metastasis (TNM) classification^2^. However, cancer staging alone does not accurately predict survival in patients with cancer; it has been shown that there are other clinicopathological factors and molecular markers that can improve prognostic estimation, such as certain genetic mutations and comorbidities^3,4^. More precise prognosis could help understand the reasons of disease progression, decide on most appropriate treatment plan, and improve survival of patients with cancer.

In an era of precision medicine, knowledge of patient characteristics and biomarkers that are associated with prognosis and survival is key for further scientific advances and meaningful improvements of cancer patient outcomes^5^. Nowadays, the majority of oncology clinical trials include biomarkers, compared to only 15% in 2000^6^. Clinical trial strategies have become more complex over time, examining multiple biomarkers per trial and exploring pan-cancer biomarkers. Yet, there is a lack of evidence base of the benefits of the use of biomarkers on patient survival^7^. Further understanding of the impact of genetic and clinicopathological factors on patient’s survival is needed to advance the field of precision cancer medicine. With sufficient evidence of the impact an individual patient’s characteristics have on cancer prognosis and survival, treatments and interventions can be focused on those patients who will benefit, sparing the side effects, treatment time and expense for those who will not.

The objective of this review is to summarise published evidence on biomarkers (clinicopathological factors and genetic mutations [SNPs]) that are associated with cancer survival in 11 major cancer types: lung, colorectal, breast, prostate, melanoma, renal, glioma, bladder, leukaemia, endometrial, ovarian. These 11 cancer types include most common cancers worldwide^8^ and are consistent with the cancer data available to the research community in one of the most widely known genomic data resources provided by Genomics England^9^. We provide two comprehensive lists of validated clinicopathological factors and genes that are important in cancer prognosis and will be valuable resources for cancer researchers, health practitioners and patients.

## Results

**Fig. 1:**
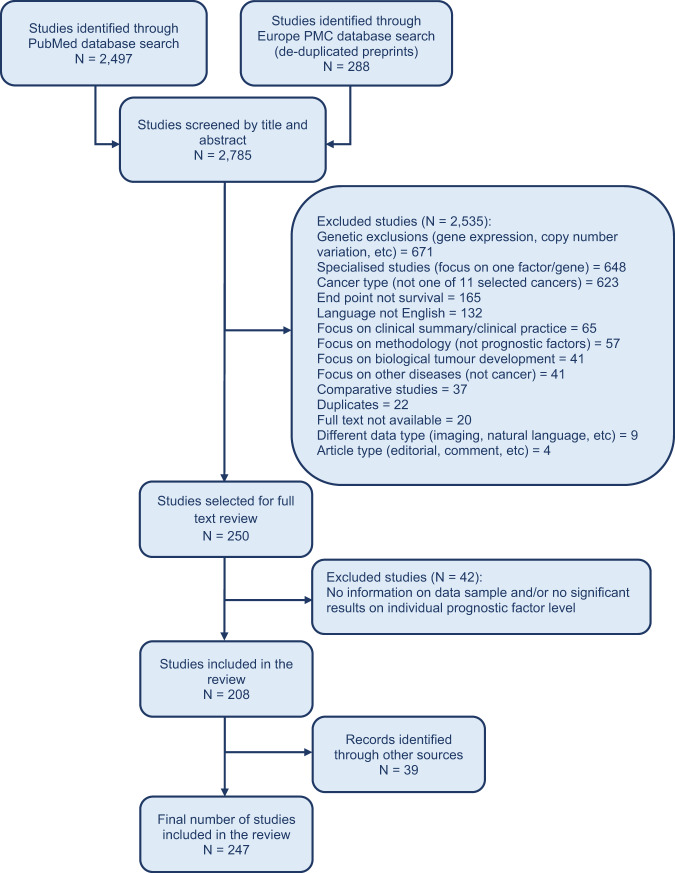
PRISMA flow diagram of study inclusions and exclusions.

The literature search identified a total of 2785 articles. 39 additional articles were identified through other sources such as reference and citation review. After title and abstract screening and removal of articles with insufficient data, 247 articles were included in the systematic review. PRISMA flow diagram is provided in Fig. 1. Full details of title and abstract screening are provided in Supplementary Data 1. Full list of references of studies included in this systematic review is provided in Supplementary References. Most of the studies were retrospective cohort studies focusing on one cancer type, 8 articles included more than one cancer type (pan-cancer studies) and 6 articles were reviews (including 2 articles that included both a review and their original data analysis). Details on selection criteria are provided in Supplementary Note 2. The number of papers excluded and reasons for exclusion are provided in Fig. 1.

Studies were categorised into three groups based on their primary focus: (1) clinical (studies conducted on hospital or similar data and primarily focusing on clinicopathological factors)—115 studies; (2) genetic (studies conducted on genetic data with the primary focus on SNPs)—119; (3) clinical and genetic (studies considering both clinicopathological factors and SNPs)—13 studies. Studies were also categorised by cancer type (Table 1) and publication year (Supplementary Table 1). An upward trend of articles published in this topic was observed with about half of articles published in the last 5 years. More than half of all publications were related to three most common cancers: lung, colorectal and breast. The lowest number of articles was observed for bladder and glioma cancers.

**Table 1:** Number of studies reviewed by cancer type.

Cancer type	Number of papers	Percentage of papers
Lung	51	20.6%
Colorectal	49	19.8%
Breast	39	15.8%
Melanoma	21	8.5%
Ovarian	15	6.1%
Prostate	14	5.7%
Leukaemia	14	5.7%
Renal	11	4.5%
Endometrial	10	4.0%
Glioma	9	3.6%
Pan-cancer	8	3.2%
Bladder	6	2.4%
Grand Total	247	100.0%

Total number of studies and percentage of studies included in the systematic review of literature up till 1^st^ July 2021, broken down by cancer type, provided in descending order. Pan-cancer studies are studies that include two or more cancer types.

The most frequently reported prognostic factors are summarised in Tables 2 and 3. Full lists of clinicopathological factors and genes recorded during the systematic review (including those recorded only in one study) are provided in Supplementary Data 2 and 3. Tables with extracted data by study are provided in Supplementary Data 4 and 5. 246 individual studies were included in the clinicopathological factor analysis representing 908,567 patients. 144 individual studies were included in the genetic mutation analysis representing 210,802 patients. The total number of individual studies is higher than the number of articles included in the systematic review due to three reasons. Firstly, studies that analysed both clinicopathological and genetic factors were included in both analyses. Secondly, pan-cancer studies which reported results by cancer type were split into multiple individual studies. Thirdly, review articles were split into multiple studies.

**Table 2:** Prognostic clinicopathological factors associated with survival in patients with cancer.

Clinicopathological factor	Risk/ Protective	Total number of patients analysed	Number of studies that found significant	Number of studies found significant in multivariable analysis	Number of studies found significant in univariable analysis	Cancer types where factor was significant
Age (age in years or age groups)	Risk	354,064	135	89	46	Breast, Colorectal, Lung, Renal, Endometrial, Ovarian, Glioma, Prostate, Melanoma, Leukaemia, Bladder, Pan-cancer
Stage (TNM or other staging system)	Risk	816,673	133	98	35	Breast, Colorectal, Lung, Renal, Endometrial, Pan-cancer, Ovarian, Glioma, Prostate, Melanoma, Leukaemia, Bladder
Histological grade	Risk	720,076	83	44	39	Breast, Colorectal, Lung, Renal, Endometrial, Pan-cancer, Ovarian, Glioma, Prostate, Bladder
Gender (male, female)	Risk	275,547	72	36	36	Breast, Colorectal, Lung, Renal, Glioma, Melanoma, Leukaemia, Bladder
Size (tumour or metastatic nodules)	Risk	32,588	52	14	38	Breast, Colorectal, Lung, Renal, Ovarian, Glioma, Melanoma
Tumour site	Risk/Protective	155,992	48	17	31	Breast, Colorectal, Lung, Renal, Melanoma
Lymph node status	Risk	18,457	41	22	19	Breast, Colorectal, Lung, Renal, Endometrial, Ovarian, Melanoma
Invasion (lymphatic, vascular, venous, perineural, myometrial)	Risk	45,723	37	21	16	Breast, Colorectal, Lung, Renal, Endometrial, Melanoma, Bladder
Subtype (e.g., ductal, lobular, mucinous or other for breast cancer)	Risk/Protective	142552	36	17	19	Breast, Lung, Renal, Endometrial, Pan-cancer, Ovarian, Glioma, Melanoma, Leukaemia, Colorectal
Chemotherapy	Protective	560,352	28	16	12	Breast, Colorectal, Lung, Ovarian, Glioma, Melanoma
Surgery	Protective	626,393	22	18	4	Breast, Colorectal, Lung, Renal, Ovarian, Prostate, Melanoma
Metastasis at diagnosis (incl. distant)	Risk	45,376	22	17	5	Breast, Colorectal, Lung, Renal, Ovarian, Melanoma
Preoperative CEA serum level	Risk	23,337	22	11	11	Colorectal, Lung
Race/Ethnicity	Risk/Protective	181,897	18	14	4	Breast, Colorectal, Lung, Ovarian, Melanoma, Leukaemia, Pan-cancer, Prostate
Smoking	Risk	559,273	16	10	6	Colorectal, Lung, Prostate, Bladder
Number of metastatic sites	Risk	20,339	15	9	6	Breast, Colorectal, Lung, Ovarian, Melanoma, Pan-cancer
ECOG patient performance status	Risk	24,445	14	12	2	Colorectal, Lung, Renal, Prostate, Melanoma, Leukaemia, Pan-cancer
Thickness (incl Breslow depth)	Risk	32,877	13	11	2	Melanoma
Number of involved lymph nodes	Risk	6310	13	4	9	Colorectal, Lung, Melanoma, Leukaemia, Breast
Radiotherapy	Protective	52,472	12	8	4	Breast, Lung, Ovarian, Glioma, Melanoma
ER (estrogen receptor) status	Protective	24,765	12	4	8	Breast, Endometrial, Colorectal
Ulceration	Risk	28,132	11	9	2	Colorectal, Melanoma
BMI/weight	Risk	32,380	11	7	4	Breast, Colorectal, Lung, Renal, Ovarian, Prostate, Bladder
KPS (Karnofsky performance status)	Protective	2448	11	6	5	Breast, Lung, Glioma, Melanoma, Pan-cancer
Bowel obstruction	Risk	8249	11	5	6	Colorectal
Comorbidities	Risk	8061	10	7	3	Breast, Colorectal, Ovarian, Melanoma, Pan-cancer
White blood cell count (WBC)	Risk	5286	10	8	2	Endometrial, Leukaemia
Education level	Protective	6186	9	6	3	Breast, Colorectal, Lung, Pan-cancer, Prostate, Ovarian
PR (progesterone receptor) status	Protective	3935	9	4	5	Breast, Endometrial
Serum lactate dehydrogenase level (LDH)	Risk	3867	9	4	5	Lung, Leukaemia, Pan-cancer, Prostate
Hepatic (liver) metastasis	Risk	3791	8	5	3	Breast, Colorectal, Lung, Prostate
Diabetes (incl. T1 and T2, with and without complications)	Risk	552,932	8	4	4	Breast, Colorectal, Lung, Renal, Endometrial, Prostate
Family history	Risk	505,217	8	4	4	Breast, Colorectal, Bladder
Marital status	Risk	94,303	7	4	3	Breast, Colorectal, Lung, Ovarian
Residual disease after surgery	Risk	1333	7	6	1	Colorectal, Ovarian, Glioma
Haemoglobin	Risk	18,544	7	5	2	Lung, Renal, Prostate, Leukaemia
Albumin	Risk/Protective	15,983	6	4	2	Breast, Lung, Prostate, Pan-cancer, Renal
Platelet count	Risk/Protective	504,457	6	3	3	Colorectal, Leukaemia, Renal
ALP (alkaline phosphatase)	Risk	1688	6	3	3	Breast, Colorectal, Lung, Pan-cancer, Prostate
Deprivation/ Socioeconomic status	Risk	536,454	5	4	1	Breast, Colorectal
COPD (incl. previous admissions for COPD)	Risk	108,555	5	4	1	Breast, Colorectal, Lung, Prostate
Lymphocyte count	Risk/Protective	3204	5	4	1	Leukaemia, Pan-cancer
Gleason score	Risk	5687	4	4	0	Prostate
Recurrence	Risk	2954	4	4	0	Breast, Colorectal, Lung
Bone metastasis	Risk	2854	4	3	1	Lung, Prostate
CA19-9 level	Risk	2747	4	3	1	Colorectal
Treatment (any vs none)	Risk	1541	4	3	1	Endometrial, Pan-cancer, Breast, Colorectal
Cognitive status	Risk	1331	4	3	1	Pan-cancer, Prostate, Colorectal
Anaemia	Risk	11,460	3	3	0	Prostate, Leukaemia
CA125 (carbohydrate antigen-125)	Risk	4798	3	3	0	Lung, Pan-cancer, Colorectal
Operative extent (radical vs partial)	Risk	6093	3	3	0	Breast, Lung, Renal

A list of prognostic clinicopathological factors associated with survival in patients with cancer, reported significant in at least 3 studies using multivariable methods. Column “Risk/Protective” indicates whether a prognostic factor is a risk or protective factor for cancer survival. Some factors can be both risk and protective, depending on cancer type. Refer to Supplementary Data 2 for a full list of clinicopathological factors, including grouping into categories and more details on prognostic direction. Column “Number of studies that found significant” is the total of columns “Number of studies that found significant in multivariable analysis” and “Number of studies that found significant in univariable analysis”. If a study reported results for both multivariable and univariable analyses, it is counted towards multivariable results only. Column “Total number of patients analysed” is a sum of sample size across all studies that found a factor significant in either multivariable or univariable analysis. Column “Cancer types where factor was significant” lists unique cancer types investigated in studies that found a factor significant.

**Table 3:** Genes with prognostic SNPs associated with survival in patients with cancer.

Gene	Gene Name	Prognostic Direction	Total number of patients analysed	Number of studies that found significant	Number of studies found significant in multivariable analysis	Number of studies found significant in univariable analysis	Cancer types where gene was significant
*BRCA2*	BRCA2, DNA repair associated	Risk/Protective	5713	11	10	1	Breast, Colorectal, Ovarian, Prostate, Bladder, Lung, Endometrial
*TP53*	Tumour protein p53	Risk	13,233	11	6	5	Breast, Colorectal, Lung, Renal, Endometrial, Leukaemia, Pan-cancer
*BRAF*	B-Raf proto-oncogene, serine/threonine kinase	Risk	4100	7	7	0	Colorectal, Melanoma
*KRAS*	Proto-oncogene, GTPase	Risk	3973	6	5	1	Colorectal
*BRCA1*	DNA repair associated	Risk/Protective	2889	6	4	2	Breast, Ovarian, Endometrial
*BIRC5*	Baculoviral IAP repeat containing 5	Risk	3543	5	5	0	Glioma, Lung, Ovarian, Melanoma, Endometrial
*ERCC2*	Excision repair 2, TFIIH core complex helicase subunit	Risk/Protective	2727	5	4	1	Colorectal, Lung, Bladder
*CDH1*	Cadherin 1	Risk/Protective	8844	4	4	0	Colorectal
*NRAS*	Neuroblastoma RAS viral oncogene homolog	Risk/Protective	3659	4	4	0	Colorectal, Melanoma, Leukaemia
*PTEN*	Phosphatase and tensin homolog	Risk/Protective	269	4	3	1	Colorectal, Endometrial
*ASXL1*	Additional sex combs like 1, transcriptional regulator	Risk	2315	3	3	0	Leukaemia
*ECD*	Ecdysoneless cell cycle regulator	Risk	11,986	3	3	0	Pan-cancer, Renal, Ovarian
*IL4R*	Interleukin 4 receptor	Risk	15,330	3	3	0	Colorectal, Prostate
*WDR82*	WD repeat domain 82	Risk	2401	3	3	0	Renal, Lung, Melanoma

A list of prognostic genes associated with survival in patients with cancer, reported significant in at least 3 studies using multivariable methods. Column “Risk/Protective” indicates whether a SNP mutation in a gene is a risk or protective factor for cancer survival. Some gene mutations can be both risk and protective, depending on cancer type. Refer to Supplementary Data 3 for a full list of genes, including identified SNP IDs. Column “Number of studies that found significant” is the total of columns “Number of studies that found significant in multivariable analysis” and “Number of studies that found significant in univariable analysis”. If a study reported results for both multivariable and univariable analyses, it is counted towards multivariable results only. Column “Total number of patients analysed” is a sum of sample size across all studies that found a gene mutation significant in either multivariable or univariable analysis. Column “Cancer types where gene was significant” lists unique cancer types investigated in studies that found a gene significant.

### Prognostic clinicopathological factors

Table 2 presents the results of clinicopathological factors associated with survival that were reported significant in at least three multivariable studies (51 factors). Supplementary Data 2 provides a full list of prognostic clinicopathological factors, including more information on effect direction. The two prognostic factors that were found significant in the majority of the studies were patient’s age (135 studies) and pathological stage (133 studies). Both age and stage were unanimously found as risk factors in all cancer types; older patients and cancers with later stage were associated with reduced survival. Similarly, histological grade was frequently found as significant prognostic factor (83 studies) with higher grade leading to shorter survival. Grade was not relevant as a prognostic factor in melanoma and leukaemia.

### Demographic factors

In addition to age, five other demographic prognostic factors were identified: gender (72 studies), race/ethnicity (18 studies), education level (9 studies), marital status (8 studies), and deprivation/socioeconomic status (5 studies). Male patients had shorter survival in breast, lung, renal, glioma, melanoma, leukaemia and bladder cancers, while colorectal cancer studies showed conflicting results. The prognostic direction of race/ethnicity depended on cancer type and population of the data sample. Higher education level, married status and higher socioeconomic status were associated with improved survival.

### Tumour-related factors

Although pathological stage and histological grade were most frequently reported tumour-related factors, 12 other tumour-related factors were often reported, including size of tumour or metastatic nodules (52 studies), tumour site (48 studies), lymph node status (41 studies), invasion (37 studies), cancer subtype (36 studies), metastasis in general (22 studies) or metastasis to specific organs such as bone metastasis (4 studies) and liver metastasis (8 studies), number of metastatic sites (15 studies) and number of involved lymph nodes (13 studies). It was noted that some of these tumour-related factors are the constituents of tumour staging or grading (e.g., tumour size or lymph node status). All these factors were identified as risk factors for survival (presence or higher number/size of analysed factors led to shorter survival) with an exception of cancer subtype and tumour site, which had a prognostic direction dependent on cancer type. Two cancer type-specific factors were identified frequently as prognostic factors for cancer survival: thickness (Breslow depth) was important in melanoma (13 studies) and Gleason score was important in prostate cancer (4 studies).

### Lifestyle and family history factors

Two lifestyle characteristics were frequently found as prognostic factors for cancer survival: smoking (16 studies), and weight/BMI (11 studies). Smoking was an important risk factor for shorter survival in lung, colorectal, prostate and bladder cancers. Higher weight/BMI was an important risk factor for shorter survival in breast, lung, ovarian, prostate and bladder cancers. However, the opposite effect of higher weight/BMI was recorded in renal and colorectal cancers. Family history was identified as an important risk factor for shorter survival in 8 studies on breast, colorectal and bladder cancers.

### Treatment-related factors

Some studies included treatment strategies in their prognostic models and majority found that surgery (22 studies), chemotherapy (28 studies) and radiotherapy (12 studies) help improve survival for patients with cancer. Operative extent (3 studies), residual disease after surgery (7 studies), whether cancer recurred (4 studies) and whether there was any treatment performed (4 studies) were also important prognostic factors for survival. Two well-known patient performance status assessment systems were frequently found as significant prognostic factors for survival: ECOG^10^ (14 studies) and KPS^11^ (11 studies).

### Factors related to other conditions

Presence or higher number of comorbidities in patients with cancer were found as important prognostic factors for reduced survival (10 studies). Some studies included comorbidities as a binary variable (whether a patient has any comorbidities or not), while others used composite comorbidity scores or grading systems (e.g., mild, moderate, severe comorbidities). In addition, individual comorbidities such as diabetes (8 studies) and chronic obstructive pulmonary disease (COPD; 5 studies) were found as significant prognostic factors for shorter survival in multiple cancer types. Bowel obstruction was an important factor leading to shorter survival in colorectal cancer (11 studies). Ulceration was an important factor for shorter survival in colorectal and melanoma cancers (11 studies). Anaemia was found significant in leukaemia and prostate cancer (3 studies). Low patient’s cognitive status was associated with shorter survival in prostate, colorectal and pan-cancer studies (4 studies).

### Clinical biomarkers

Several clinical biomarkers were identified as important prognostic factors for cancer survival. In colorectal and lung cancers, higher CEA serum level was associated with shorter survival (22 studies). ER (estrogen receptor) (12 studies) and PR (progesterone receptor) (9 studies) status were important prognostic factors in breast and endometrial cancers (ER status was also important in colorectal cancer). ER and PR positive patients were found to survive longer in all studies. Higher white blood cell count was an important risk factor in leukaemia and endometrial cancer leading to shorter survival (10 studies). Elevated serum lactate dehydrogenase (LDH) level was associated with shorter survival in lung, leukaemia, prostate cancers and on pan-cancer level (9 studies). Decreased haemoglobin was associated with reduced survival in lung, renal, prostate and leukaemia cancers (7 studies). High alkaline phosphatase (ALP) level was associated with shorter survival in breast, colorectal, lung, prostate cancers and on pan-cancer level (6 studies). Albumin level, platelet count and lymphocyte count were also associated with survival, but the effect direction differed by cancer type. In colorectal cancer studies, higher CA19-9 (carbohydrate antigen 19-9) (4 studies) and CA125 (carbohydrate antigen-125) (1 study) levels were associated with shorter survival. CA125 was also found as a significant risk factor for survival in lung cancer and pan-cancer studies (2 studies). Although CA125 is used as a marker for treatment response in ovarian cancers in clinical practice, only one study in our review showed significant association between CA125 and ovarian cancer (pan-cancer study which included ovarian and endometrial cancers). All 15 studies which focused on ovarian cancer either did not test or did not find CA125 to be significantly associated with overall survival.

### Prognostic gene mutations

Table 3 presents the results of genes associated with survival that were recorded as significant in at least three multivariable studies (14 genes). *TP53* gene mutation was one of the most frequently reported prognostic risk factors for cancer survival (11 studies). *TP53* gene encodes a tumour suppressor protein that plays an important role in cell cycle arrest, apoptosis, senescence, DNA repair and changes in metabolism and is widely known to be associated with cancer disease^12^. The review found that *TP53* was an important prognostic risk factor in colorectal, lung, renal, endometrial, leukaemia cancers and on pan-cancer level. In breast cancer, the effect direction depended on cancer sub-type. Two studies found that *TP53* mutations were associated with worse outcome in patients with ER positive status, but not in patients with ER negative status. *PTEN* gene is another tumour suppressor gene, for which mutations have been observed in many cancers^13^. Mutations in *PTEN* gene were associated with shorter survival in colorectal cancer, but longer survival in endometrial cancer (4 studies).

Two DNA repair genes, *BRCA1* and *BRCA2* were among the most frequently reported genes to be associated with survival (6 and 11 studies respectively). *BRCA1* and *BRCA2* genes encode proteins that help maintain genomic stability and act as tumour suppressors. *BRCA* mutations in the germline have become a hallmark for hereditary breast and ovarian cancers^14^. In this systematic review it was found that the effect direction of mutations in *BRCA1* and *BRCA2* genes varies by cancer type. Mutations in *BRCA1* gene were associated with shorter survival in breast cancer, but longer survival in ovarian and endometrial cancers. Mutations in *BRCA2* gene were associated with shorter survival in breast, colorectal and bladder cancers, but longer survival in ovarian, endometrial and lung cancers. *ERCC2* was another gene related to DNA repair (nucleotide excision repair pathway), that was associated with cancer survival (5 studies). *ERCC2* mutations were associated with shorter survival in colorectal and lung cancer, but were found to prolong survival in bladder cancer.

Mutations in oncogenes *BRAF*, *KRAS* and *NRAS* were frequently observed as prognostic factors for shorter survival (7, 6 and 4 studies respectively). RAS family oncogenes *KRAS* and *NRAS* have long been known to be associated with cancer^15^, and along with *BRAF* oncogene were frequently found in colorectal cancer^16,17^. In this review, all three oncogenes were associated with shorter survival in colorectal cancer. *BRAF* and *NRAS* gene mutations were also associated with shorter survival in melanomas. *NRAS* gene was found to be a protective prognostic factor in one study on leukaemia.

*BIRC5*, an immune-related gene, is a member of the inhibitor of apoptosis gene family, which encode negative regulatory proteins that prevent cell death and promote cell proliferation. It was shown to be highly expressed and lead to poor prognosis in most cancers previously^18^. In this review, *BIRC5* gene mutations have been associated with shorter survival in glioma, lung, ovarian, melanoma and endometrial cancers (5 studies).

*CDH1* gene is most commonly known to predispose diffuse gastric and lobular breast cancers^19^. It encodes cadherin protein and loss of function in this gene contributes to cancer progression by increased proliferation, invasion and metastasis. In this review, *CDH1* gene was identified as significant prognostic factor for overall survival in colorectal cancer with some studies reporting risk while others protective effect (4 studies).

*ASXL1* gene is known to be frequently mutated in all types of malignant myeloid diseases including chronic myelomonocytic leukaemia and acute myeloid leukaemia. *ASXL1* mutations are most frequent in chronic myelomonocytic leukaemia (about 45%).^20^ This review found *ASXL1* mutations to have a prognostic effect on survival in leukaemia. Three studies reported *ASXL1* mutations to be associated with shorter overall survival in chronic myelomonocytic and acute myeloid leukaemias.

*ECD*, *IL4R* and *WDR82* are protein coding genes, not commonly known to be associated with cancer risk or prognosis. However, multiple studies in this review identified mutations in these genes as prognostic factors for cancer survival. *ECD* mutations were found to be associated with shorter survival in renal, ovarian and pan-cancer studies (3 studies). *IL4R* mutations were found to be associated with shorter survival in colorectal and prostate cancers (3 studies). *WDR82* mutations were found to be associated with shorter survival in renal, lung and melanoma cancers (3 studies).

## Discussion

We extracted and summarised data on clinicopathological and genetic factors associated with survival from 247 articles across eleven cancer types. The findings indicate that prognostic factors for cancer survival have been investigated previously, however usually focusing on one cancer type and/or one data type. This summary of previously published literature shows that there is more diversity in research conducted on genetic prognostic factors related to cancer survival when compared to clinicopathological factors. Even though a large number of genetic mutations were identified to be associated with cancer survival (440 genes), only 40 of these were found significant in more than one multivariable study, and only 14 in three or more studies. In contrast, there were fewer clinicopathological prognostic factors identified overall (238 factors), but 79 of these were found significant in more than one multivariable study, and 51 were found significant in three or more studies. This is not surprising due to the differences between clinicopathological and genetic data that is collected from patients with cancer. The clinical, pathological and demographic information collected about the patient and the tumour are usually limited to tens or hundreds of data points, while the size of genetic data is substantially bigger, making genetic mutation analysis on whole-genome level difficult and time consuming. Therefore, most genetic studies focus on one biological pathway or group of genes associated with cancer leading to less overlap across genetic studies.

Our findings were in line with a previously published systematic review on clinicopathological prognostic factors in patients with incurable cancer^21^, which found comorbidities, gender, tumour site, tumour bulk, metastasis and performance status to be associated with survival. In addition, our review identified patient’s age and stage as the most frequently reported factors associated with survival. Considering the review cited above focused on patients with incurable cancer, they all would have had cancer with advanced stage; hence stage was not reported among prognostic factors. Age was among the most frequently tested prognostic factors in the aforementioned review, but results on the effect direction of age were inconsistent across studies some showing better survival for younger and some for older patients. In our review, older age was found to be consistently associated with reduced survival.

Despite the emergence of personalised cancer treatments in the recent years, there is still a gap between prognostic biomarker discoveries and their clinical use. Healthcare providers do not feel adequately informed about the evidence of the biomarker association to patient outcomes^22^. This review provides a unique view of clinicopathological and genetic prognostic factors across different cancer types and large patient base. The findings confirm that factors such as age, tumour stage, size and grade, tumour spread to other organs can be used to predict cancer survival. A lot of these factors are well known and widely used in clinical practice for prognosis and treatment of cancer. However, this overview draws attention to other, less commonly used, factors, which might help produce more precise prognosis and survival estimates in the future. Biomarkers such as CEA serum level, haemoglobin level, albumin level, white blood cell count, serum lactate dehydrogenase level, platelet count, alkaline phosphatase, lymphocyte count, CA19-9 level and CA125 level were found to be significantly associated with overall survival and could be collected and used as objective markers in future work on cancer prognosis. This overview demonstrates the prognostic ability of SNP mutations in well-known genes such as *TP53*, *BRCA1*, *BRCA2*, *BRAF*, *KRAS* and less commonly known genes such as *ECD*, *IL4R* and *WDR82*.

The information presented in this systematic review contributes to the understanding of cancer disease and could be used by researchers to further test and build the knowledge base about prognostic factors for cancer survival. This information could be used to develop complex prognostic models, which in turn could help predict cancer prognosis more accurately. Most importantly, this information could be used in the design of biomarker-driven oncology clinical trials^23,24^, which might lead into discoveries of new cancer treatments.

This systematic review summarises clinicopathological and genetic prognostic factors related to cancer survival at a large scale and on pan-cancer level. The results presented in this review are based on a high number of studies with large patient samples (average sample size in clinicopathological studies was 3,693 patients, and 1,464 patients in genetic studies). In addition to summarising widely known prognostic factors for cancer survival, our review draws attention to a number of less known factors, which haven’t been commonly used for cancer prognosis in research and clinical practice. Conducted on pan-cancer level, our review allows for comparison across different cancers and detects prognostic biomarkers that are important across multiple cancer types.

We acknowledge several limitations. This review was completed on a large scale and therefore provides summarised aggregate information about prognostic factors associated with cancer survival. For in depth understanding of factor effect sizes, a more specialised review should be completed, including a meta-analysis. Due to the large scale of this review, only the factors that were found significant in at least one study were included. However, information about insignificant factors could be useful for certain use cases and could be collected in a review of a smaller scale (e.g., in a review focusing on one cancer type). Due to the lack of detail and inconsistent reporting of insignificant results in included studies, only significant results were summarised in this review. Distinction between significance in multivariable and univariable analyses provides information on which factors remain significantly associated with cancer survival after inclusion of other important prognostic factors.

Single nucleotide polymorphisms were the focus of the genetics part of this review, however a similar summary could be conducted for other types of genetic variation such as copy number variation, which would help build even better understanding about the genes and their functions’ prognostic association with cancer survival. We combined the findings about genetic prognostic variables from both germline and somatic mutations, however it should be noted that these mutations could bear different meaning for cancer prognosis and treatment possibilities. Targeted cancer therapies due to the finding of known predictive biomarkers and genetic factors were not in scope of this review, but they have been increasingly used in practice in recent years and are known to significantly affect survival outcomes. Further studies could be performed with a focus on treatment-related prognostic effects on cancer survival.

We focused on individual prognostic factors associated with survival, because interaction effects were rarely reported in the literature. Exploring prognostic effects of different factor interactions (including cross-sectional interactions between clinicopathological and genetic factors) could be the focus of future research. The findings show that there is more research completed for more common cancer types such as lung, breast or colorectal cancers, however the evidence base for less common cancers like glioma or bladder cancer is slim. More research should be done on less common cancers in order to improve the knowledge, prognosis prediction and availability of personalised treatments for these cancers. The amount of healthcare data being collected is increasing exponentially, which has led to the rise of application of machine learning and similar computational methods in healthcare. However, this review shows that the majority of research conducted on prognostic factors related to cancer survival are based on traditional regression methods, such as Cox proportional hazards regression. Only 6 out of 247 articles used a machine learning approach (e.g., random forest or neural networks). The use of machine learning methods in the future might help assess larger amounts of data and develop insights on a larger scale.

This systematic review of 247 studies found 440 genes, including *TP53*, *BRCA1*, *BRCA2*, *BRAF*, *KRAS*, *BIRC5*, and 238 clinicopathological factors, including patient’s age and gender, tumour stage, histological grade, size, site, subtype, invasion and lymph node status, that are important prognostic factors for cancer survival. A summary of this scale helps improve understanding of prognostic factors across different cancer types and shows the gaps in research such as prognostic effects of interactions and less common cancer types. It also serves as a knowledge base for biomarker-driven oncology trials and further research work on cancer prognostic models, which both could lead to improvements in patient care and outcomes.

## Methods

### Information sources and search

This systematic review was conducted and reported according to The Preferred Reporting Items for Systematic Reviews and Meta-analyses (PRISMA) guidelines^25^. A literature search was conducted using PubMed/MEDLINE and Europe PMC from database inception up until 1^st^ July 2021. The search strategy contained inclusion and exclusion criteria described in free text words as well as database-specific terms (MeSH terms in PubMed/MEDLINE and keywords in Europe PMC). No limit for publication dates was applied. Full details of search inclusion and exclusion criteria as well as reproducible search terms for both databases are provided in Supplementary Note 1. All published literature found in PubMed/MEDLINE database were included in the review with an addition of de-duplicated preprints from Europe PMC database to capture most recent studies in this topic. The references and citations of key studies were reviewed, and additional articles were added to the review.

### Study selection

Search criteria and study selection criteria were reviewed together and agreed by both authors. Any disagreements between reviewers were resolved through discussion. Titles and abstracts were reviewed first, and full text was retrieved for those studies where decision could not be made based on the abstract. Eligibility and exclusion criteria used in the screening of titles and abstracts are provided in Supplementary Note 2. Remaining articles were reviewed in full text and excluded from the final review if an article was a review and did not provide sufficient data about prognostic factors (sample size, factor significance, effect direction and/or univariable/multivariable method) and/or an article did not report significant associations with survival on individual factor level.

### Data extraction

Full text and supplementary materials of selected studies were reviewed to extract the data. Effects of prognostic factors and genetic mutations were synthesised by vote counting and summarising the direction of the effect^26^. The direction of the effect was determined by analysing reported effect sizes. It was noted whether a reported effect (e.g., hazard ratio) showed association to reduced survival (risk factor) or improved survival (protective factor). Direction of reported effects associated with patient survival was extracted if they reached significance of *p*-value <0.05. The effects from univariable and multivariable analyses were summarised separately. When either univariable or multivariable results were reported in a study, they were extracted and counted towards their respective category. When a factor was significant in both multivariable and univariable analyses in a single study, multivariable analysis took priority for the result extraction. When a factor was found significant in univariable analysis, but not in multivariable analysis (or it was not tested in multivariable analysis), only univariable result was extracted. In rare cases, where it was not clear whether reported results are from univariable or multivariable analysis, the results were not extracted. For each individual factor, the effect direction was recorded including notes on conflicting results. If there were conflicting results, the notes were taken focusing on differences across cancer types. A factor was included in the summary if at least one study recorded its effect direction.

The main end point for data collection was overall survival of patients with cancer. If overall survival was not reported, effects related to cancer specific, disease free, progression free, event free or time specific survival were collected. Review articles were included in the synthesis if they provided sample size and significance of prognostic effects reported in the reviewed studies. If there was an overlap between studies included in review articles and studies selected during our literature search, we included those studies as individual articles and excluded them from review article results to avoid double counting.

Two summary tables were prepared as a result of data synthesis: one for clinicopathological factors and one for genetic mutations. Some studies contributed to only one of the summary tables, while other studies were included in both. For example, some genetic studies included clinicopathological variables in the regression models for adjustment purposes. If such studies presented effects of these adjustment variables and their significance, this information was recorded in the summary of clinicopathological factors. Same rule was applied in the reverse situation (where clinicopathological studies used genetic factors for adjustment purposes), however it was rare.

Pan-cancer studies were summarised in two different ways. If a study presented results for all included cancer types overall, results were considered as one study and they were categorised as pan-cancer rather than individual cancer types. On the other hand, if a study reported results separately for cancer types, these were reported as separate studies (sample size was split accordingly). Similarly, studies included in the review articles were reported as separate studies.

Genetic studies were summarised on gene level, however specific SNP IDs were also collected if they were mentioned in the article. Both germline and somatic mutations were included in this review. Some studies did not explicitly report whether they analysed germline or somatic DNA data, while other studies analysed both types. Therefore, results in this review are summarised for germline and somatic mutations together. Clinicopathological factor summary includes all factors that were reported in reviewed articles except factors related to treatments and therapies specific to cancer type (e.g., specific drug treatment). Results about general treatments such as chemotherapy, radiotherapy, surgery are included in the results. Very few studies recorded interaction effects associated with cancer survival. These interaction variables were often specific to the study and its data sample, and did not appear in any other studies, therefore they were not included in the results. On rare occasions, where multiple studies were published using the same data sample, the results were included as multiple studies only if prognostic factors being tested were different.

### Reporting summary

Further information on research design is available in the Nature Research Reporting Summary linked to this article.

### Supplementary information

Supplementary Data 1

Supplementary Data 2

Supplementary Data 3

Supplementary Data 4

Supplementary Data 5

REPORTING SUMMARY

Supp Info

## Data Availability

All the data collected and analysed in this study are provided in supplementary materials.
