# Supplementary material for: Pan-cancer prognostic genetic mutations and clinicopathological factors associated with survival outcomes: a systematic review
Source: NPJ Precis Oncol. 2022 Apr 20;6:27. doi: 10.1038/s41698-022-00269-5 (PMC9021198; doi:10.1038/s41698-022-00269-5)
Supplement: Supplementary file 7 — Supp Info [file 41698_2022_269_MOESM7_ESM.pdf]

Supplementary Note 1. Search inclusion/exclusion criteria and reproducible search terms

Supplementary Note 2. Study selection criteria

Supplementary Table 1. Number of studies reviewed by publication year

Supplementary Data 1. Title and abstract screening results

Supplementary Data 2. Full list of prognostic clinicopathological factors associated with survival in cancer patients

Supplementary Data 3. Full list of prognostic genes associated with survival in cancer patients

Supplementary Data 4. Extracted data on prognostic clinicopathological factors associated with survival in cancer patients per study

Supplementary Data 5. Extracted data on prognostic genes associated with survival in cancer patients per study

Supplementary References. References of studies included in the systematic review

Supplementary Note 1: search inclusion/exclusion criteria and reproducible search terms

Search inclusion criteria:

1. Cancer studies. Cancer and synonym terms (e.g., neoplasm, tumour, oncology, malignancy), specific names of cancer types (e.g., melanoma, glioma, carcinoma).
2. Human studies. This criterion was written using double exclusion approach (focused on excluding animals rather than including humans) in order to avoid losing articles about humans that are new and have not been specifically marked as human studies yet.
3. Genetics and/or clinical data. Studies that used clinical data (e.g., electronic health records) or genomics/genetics data.
4. Computational methods. Studies that conducted assessment of the association of prognostic factors with patient survival. E.g., methods such as logistic regression, Cox proportional hazards, machine learning, genome wide association studies (GWAS). This criterion prevented inclusion of descriptive studies that focus on cancer statistics.
5. Prognostic factors. Studies that focused on identifying prognostic factors and genetic mutations associated with cancer prognosis and survival. The terms included prognosis, staging, grading, survival, risk/prognostic factor/feature, variant, mutation, polymorphism, signature and other synonyms.

Search exclusion criteria:

1. Diagnostic imaging. Studies that focused on machine learning and other methods to analyse diagnostic images.
2. Genetics areas. Studies from broader areas of genetics such as genetic testing, genotyping techniques, pedigrees were excluded.
3. Drugs and therapeutics. Studies that focused on assessing effectiveness of a drug treatment, comparing different therapeutic strategies or assessing the dosage and adverse effects of medications were excluded.

Full search terms and query for PubMed/MEDLINE database:

((neoplasms[MeSH] OR cancer*[Title] OR "pan-cancer*"[Title] OR pancancer[Title] OR carcinoma*[Title] OR tumor*[Title] OR tumour*[Title] OR malignan*[Title] OR neoplasm*[Title] OR adenocarcinoma*[Title] OR leukemia*[Title] OR leukaemia*[Title] OR melanoma*[Title] OR glioma*[Title] OR glioblastoma*[Title] OR onco*[Title]) NOT ("organisms category"[MeSH] NOT "humans"[MeSH]) NOT ("mice"[MeSH]) AND ("genetics"[MeSH] OR "genomics"[MeSH] OR genetic*[Title/Abstract] OR genom*[Title/Abstract] OR "medical records"[MeSH] OR "hospital records"[MeSH] OR ((clinical[Title/Abstract] OR medical[Title/Abstract] OR health[Title/Abstract] OR hospital[Title/Abstract]) AND (record*[Title/Abstract] OR data[Title/Abstract])) OR "secondary care"[Title/Abstract] OR "primary care"[Title/Abstract] OR "general practice*"[Title/Abstract] OR "GP"[Title/Abstract]) AND ("regression analysis"[MeSH] OR "regression*"[Title/Abstract] OR "logistic"[Title/Abstract] OR "proportional hazard*"[Title/Abstract] OR "cox"[Title/Abstract] OR "multivariate analysis"[MeSH] OR "multivariate*"[Title/Abstract] OR "multivariable*"[Title/Abstract] OR "principal component analysis"[MeSH] OR "principal component analys*"[Title/Abstract] OR "machine learning"[MeSH] OR "machine learning"[Title/Abstract] OR "deep learning"[Title/Abstract] OR "decision trees"[Mesh] OR "decision tree*"[Title/Abstract] OR "random forest*"[Title/Abstract] OR "neural networks, computer"[Mesh] OR "neural network*"[Title/Abstract] OR "genetic association studies"[MeSH] OR GWAS[Title/Abstract] OR ((genetic*[Title/Abstract] OR genom*[Title/Abstract]) AND (association[Title/Abstract])) OR "Sequence Analysis, DNA"[MeSH] OR ((sequence*[Title/Abstract] OR mutation*[Title/Abstract]) AND (analysis[Title/Abstract])) OR "review"[Publication Type] OR "systematic review"[Publication Type] OR "meta-analysis"[Publication Type] OR review[Title/Abstract] OR "meta-analysis"[Title/Abstract]) AND("neoplasm grading"[MeSH] OR "neoplasm staging"[MeSH] OR "disease-free survival"[Mesh] OR "progression-free survival"[Mesh] OR prognos*[Title] OR stage*[Title] OR staging*[Title] OR grade*[Title] OR grading*[Title] OR survival[Title]) AND ("risk factors"[MeSH] OR "risk assessment"[MeSH] OR "genetic variation"[MeSH] OR (risk*[Title] AND (factor*[Title] OR feature*[Title])) OR (prognos*[Title] AND (factor*[Title] OR feature*[Title])) OR signature*[Title] OR mutation*[Title] OR variant*[Title] OR polymorphism*[Title])) NOT ("diagnostic imaging"[MeSH] OR "image processing, computer-assisted"[MeSH] OR imag*[Title/Abstract] OR scan*[Title/Abstract] OR radiomic*[Title/Abstract] OR tomograph*[Title/Abstract] OR radiograph*[Title/Abstract] OR mammograph*[Title/Abstract] OR "histology"[MeSH] OR histol*[Title/Abstract] OR histopathol*[Title/Abstract] OR cytol*[Title/Abstract] OR "cytogenetics"[MeSH] OR cytogenetic*[Title/Abstract] OR "genetics, population"[Mesh] OR ("genetic techniques"[MeSH] NOT ("genetic association studies"[MeSH] OR "Sequence Analysis, DNA"[Mesh])) OR "genetic testing*"[Title/Abstract] OR "clinical trials as topic"[MeSH] OR "clinical

trial*"[Title/Abstract] OR ("chemicals and drugs category"[MeSH] NOT ("genetic markers"[MeSH])) OR "administration and dosage" [Subheading] OR "therapeutics"[MeSH] OR "surgical procedures, operative"[Mesh] OR therap*[Title] OR chemotherap*[Title] OR immunotherap*[Title] OR radiotherap*[Title] OR surger*[Title] OR operat*[Title])

Full search terms and query for Europe PMC database:

((KW:neoplasms OR TITLE:cancer* OR TITLE:pan-cancer* OR TITLE:pancancer OR TITLE:carcinoma* OR TITLE:tumor* OR TITLE:tumour* OR TITLE:malignan* OR TITLE:neoplasm* OR TITLE:adenocarcinoma* OR TITLE:leukemia* OR TITLE:leukaemia* OR TITLE:melanoma* OR TITLE:glioma* OR TITLE:glioblastoma* OR TITLE:onco*) NOT (KW:animals NOT KW:humans) NOT (KW:mice) AND (KW:genetics OR KW:genomics OR TITLE:genetic* OR ABSTRACT:genetic* OR TITLE:genom* OR ABSTRACT:genom* OR KW:”medical records” OR KW:”hospital records” OR ((TITLE:clinical OR ABSTRACT:clinical OR TITLE:medical OR ABSTRACT:medical OR TITLE:health OR ABSTRACT:health OR TITLE:hospital OR ABSTRACT:hospital) AND (TITLE:record* OR ABSTRACT:record* OR TITLE:data OR ABSTRACT:data)) OR TITLE:”secondary care” OR ABSTRACT:”secondary care” OR TITLE:”primary care” OR ABSTRACT:”primary care” OR TITLE:”general practice” OR ABSTRACT:”general practice” OR TITLE:GP OR ABSTRACT:GP) AND (KW:”regression analysis” OR TITLE:regression* OR ABSTRACT:regression* OR TITLE:logistic OR ABSTRACT:logistic OR TITLE:“proportional hazard*” OR ABSTRACT:”proportional hazard*” OR TITLE:cox OR ABSTRACT:cox OR KW:”multivariate analysis” OR TITLE:multivariate* OR ABSTRACT:multivariate* OR TITLE:multivariable* OR ABSTRACT:multivariable* OR KW:”principal component analysis” OR TITLE:“principal component analysis” OR ABSTRACT:” principal component analysis” OR KW:”machine learning” OR TITLE:“machine learning” OR ABSTRACT:”machine learning” OR TITLE:“deep learning” OR ABSTRACT:”deep learning” OR KW:”decision trees” OR ((TITLE:decision OR ABSTRACT:decision) AND (TITLE:tree* OR ABSTRACT:tree*)) OR TITLE:“random forest” OR ABSTRACT:”random forest” OR KW:”neural networks, computer” OR ((TITLE:neural OR ABSTRACT:neural) AND (TITLE:network* OR ABSTRACT:network*)) OR KW:”genetic association studies” OR TITLE:GWAS OR ABSTRACT:GWAS OR ((TITLE:genetic* OR ABSTRACT:genetic* OR TITLE:genom* OR ABSTRACT:genom*) AND (TITLE:association OR ABSTRACT:association)) OR KW:”sequence analysis” OR TITLE:”mutation analysis” OR ABSTRACT:”mutation analysis” OR TITLE:”sequence analysis” OR ABSTRACT:”sequence analysis” OR PUB_TYPE:”review” OR TITLE:review OR ABSTRACT:review OR PUB_TYPE:”meta-analysis” OR TITLE:meta-analysis OR ABSTRACT:meta-analysis) AND (KW:”prognosis” OR KW:”survival” OR TITLE:prognos* OR TITLE:stage* OR TITLE:staging* OR TITLE:grade* OR TITLE:grading* OR TITLE:survival) AND (KW:”risk factors” OR KW:”risk assessment” OR KW:”genetic variation” OR (TITLE:risk* AND (TITLE:factor* OR TITLE:feature*)) OR (TITLE:prognos* AND (TITLE:factor* OR TITLE:feature*)) OR TITLE:signature* OR TITLE:mutation* OR TITLE:variant* OR TITLE:polymorphism*)) NOT (KW:imaging OR KW:”diagnostic imaging” OR KW:”image processing, computer-assisted” OR TITLE:imag* OR ABSTRACT:imag* OR TITLE:scan* OR ABSTRACT:scan* OR

TITLE:radiomic* OR ABSTRACT:radiomic* OR TITLE:tomograph* OR ABSTRACT:tomograph* OR TITLE:radiograph* OR ABSTRACT:radiograph* OR TITLE:mammograph* OR ABSTRACT:mammograph* OR KW:histology OR TITLE:histol* OR ABSTRACT:histol* OR TITLE:histopathol* OR ABSTRACT:histopathol* OR TITLE:cytol* OR ABSTRACT:cytol* OR KW:cytogenetics OR TITLE:cytogenetic* OR ABSTRACT:cytogenetic* OR KW:"genetics, population" OR KW:"chromosome mapping" OR KW:pedigree OR KW:"genetic testing" OR TITLE:"genetic testing" OR ABSTRACT:"genetic testing" OR KW:"clinical trials" OR TITLE:"clinical trial" OR ABSTRACT:"clinical trial" OR KW:drugs OR KW:complications OR KW:"adverse effects" OR KW:"dosage" OR KW:"therapeutic" OR KW:"therapy" OR KW:"surgical procedures" OR KW:"surgery" OR TITLE:therap* OR TITLE:chemotherap* OR TITLE:immunotherap* OR TITLE:radiotherap* OR TITLE:surger* OR TITLE:operat*)

Supplementary Note 2: study selection criteria

Study selection criteria (eligibility and exclusions) applied for screening titles and abstracts:

1. Articles must focus on cancer rather than other diseases.
2. Articles must focus on survival end point/outcome rather than other end point/outcome (e.g., articles on prediction of recurrence or metastasis were excluded).
3. Genetic articles must focus on SNPs rather than other genetic data/processes (e.g., articles on gene expression, copy number variation were excluded).
4. Articles must analyse multiple prognostic factors or genetic mutations rather than have a specialised focus on assessing one factor.
5. Articles must analyse one or more of the eleven cancer types (lung, colorectal, breast, prostate, melanoma, renal, glioma, bladder, leukaemia, endometrial, ovarian), including pan-cancer studies.
6. Articles must be written in English.
7. Articles focusing on clinical summaries and clinical practice guidelines were excluded.
8. Articles without evidence-based data, such as case reports, were excluded.
9. Articles focusing on methodology (e.g., comparison of different prognostic models), where they do not provide results on individual prognostic factor level, were excluded.
10. Articles focusing on describing biological processes (e.g., biological tumour development) were excluded.
11. Specialised comparative studies comparing cancer statistics or factor effect in different populations were excluded.
12. Duplicate articles were excluded.
13. Articles that did not have full text available for review were excluded.
14. Articles focusing on diagnostic imaging or natural language processing were excluded.
15. Articles with article type such as editorial, comment or protocol were excluded.

Supplementary Table 1: number of studies reviewed by publication year

Publication year	Number of papers	Percentage of papers
<2000	17	6.9%
2000-2005	16	6.5%
2006-2010	22	8.9%
2011	8	3.2%
2012	13	5.3%
2013	15	6.1%
2014	14	5.7%
2015	13	5.3%
2016	15	6.1%
2017	17	6.9%
2018	14	5.7%
2019	23	9.3%
2020	31	12.6%
2021*	29	11.7%
Grand Total	247	100.0%

Total number of studies and percentage of studies included in the systematic review of literature up till 1st July 2021, broken down by publication year. *Only first half of the year 2021 is included in this review.

Supplementary References: references of studies included in the systematic review

1. Aas K, Axcrona K, Kvåle R, et al. Ten-year Mortality in Men With Nonmetastatic Prostate Cancer in Norway. *Urology*. 2017;110:140-147. doi:10.1016/j.urology.2017.07.048
2. Abulí A, Lozano JJ, Rodríguez-Soler M, et al. Genetic susceptibility variants associated with colorectal cancer prognosis. *Carcinogenesis*. 2013;34(10):2286-2291. doi:10.1093/carcin/bgt179
3. Adams BE, Peng PD, Williams ML. Melanoma of the Foot Is Associated With Advanced Disease and Poorer Survival. *The Journal of Foot and Ankle Surgery*. 2018;57(1):52-55. doi:10.1053/j.jfas.2017.07.018
4. Ahiko Y, Shida D, Nakamura Y, et al. Preoperative Nutritional Scores as Host-Related Prognostic Factors for Both Overall Survival and Postoperative Complications in Stage II – III Colorectal Cancer Patients. *Diseases of the Colon & Rectum*. 2021;Publish Ahead of Print. doi:10.1097/DCR.0000000000002033
5. Ahn SW, Na AS, Yang JD, Hwang HP, Yu HC, Cho BH. Scoring of prognostic factors that influence long-term survival in patients with hepatic metastasis of colorectal cancer. *Korean J Hepatobiliary Pancreat Surg*. 2011;15(3):146. doi:10.14701/kjhbps.2011.15.3.146
6. Al-Naggar RA, Isa ZM, Shah SA, et al. Eight year survival among breast cancer Malaysian women from University Kebangsaan Malaysia Medical Centre. Asian Pac J Cancer Prev. 2009;10(6):1075-1078.
7. Albert A, Lee A, Allbright R, Vijayakumar S. Vulvar melanoma: an analysis of prognostic factors and treatment patterns. *J Gynecol Oncol*. 2020;31(5):e66. doi:10.3802/jgo.2020.31.e66
8. Alimohammadi E, Bagheri SR, Sadeghsalehi A, Rizevandi P, Rezaie Z, Abdi A. Prognostic factors in patients with glioblastoma multiforme: focus on the pathologic variants. *Acta Neurol Belg*. 2020;120(6):1341-1350. doi:10.1007/s13760-019-01171-x
9. Alotaibi RM, Rezk HR, Juliana CI, Guure C. Breast cancer mortality in Saudi Arabia: Modelling observed and unobserved factors. Ahmad A, ed. *PLoS ONE*. 2018;13(10):e0206148. doi:10.1371/journal.pone.0206148
10. Andersson AP, Gottlieb J, Drzewiecki KT, Hou-Jensen K, Sondergaard K. Skin melanoma of the head and neck: Prognostic factors and recurrence-free survival in 512 patients. *Cancer*. 2010;69(5):1153-1156. doi:10.1002/cncr.2820690515
11. Ansell D, Whitman S, Lipton R, Cooper R. Race, income, and survival from breast cancer at two public hospitals. *Cancer*. 1993 Nov 15;72(10):2974-8. doi: 10.1002/1097-0142(19931115)72:10<2974::aid-cncr2820721019>3.0.co;2-m. PMID: 8221564.

12. Antoni D, Clavier J-B, Pop M, Benoît C, Lefebvre F, Noël G. An Institutional Retrospective Analysis of 93 Patients with Brain Metastases from Breast Cancer: Treatment Outcomes, Diagnosis-Specific Prognostic Factors. *IJMS*. 2012;13(12):16489-16499. doi:10.3390/ijms131216489
13. Asgari MM, Shen L, Sokil MM, Yeh I, Jorgenson E. Prognostic factors and survival in acral lentiginous melanoma. *Br J Dermatol*. 2017;177(2):428-435. doi:10.1111/bjd.15600
14. Ayremlou P, Razavi SM, Solaymani-Dodaran M, Vakili M, Asadi-Lari M. Demographic and Prognostic Factors of 455 Patients with Acute Leukemia Admitted to Two Referral Hospitals in Tehran-Iran During Ten Years (2001-2011). *Iranian Journal of Cancer Prevention*. 2012;5(3):157-163.
15. Azzato EM, Driver KE, Lesueur F, et al. Effects of common germline genetic variation in cell cycle control genes on breast cancer survival: results from a population-based cohort. *Breast Cancer Res*. 2008;10(3):R47. doi:10.1186/bcr2100
16. Bäcklund LM, Nilsson BR, Liu L, Ichimura K, Collins VP. Mutations in Rb1 pathway-related genes are associated with poor prognosis in Anaplastic Astrocytomas. *Br J Cancer*. 2005;93(1):124-130. doi:10.1038/sj.bjc.6602661
17. Bai X, Kong Y, Chi Z, et al. *MAPK* Pathway and *TERT* Promoter Gene Mutation Pattern and Its Prognostic Value in Melanoma Patients: A Retrospective Study of 2,793 Cases. *Clin Cancer Res*. 2017;23(20):6120-6127. doi:10.1158/1078-0432.CCR-17-0980
18. Balch CM, Soong S-J, Gershenwald JE, et al. Prognostic Factors Analysis of 17,600 Melanoma Patients: Validation of the American Joint Committee on Cancer Melanoma Staging System. *JCO*. 2001;19(16):3622-3634. doi:10.1200/JCO.2001.19.16.3622
19. Ban WH, Yeo CD, Han S, et al. Impact of smoking amount on clinicopathological features and survival in non-small cell lung cancer. *BMC Cancer*. 2020;20(1):848. doi:10.1186/s12885-020-07358-3
20. Bănescu C, Tripon F, Trifa AP, et al. Presence of copy number aberration and clinical prognostic factors in patients with acute myeloid leukemia: an analysis of effect modification. *Polish Archives of Internal Medicine*. Published online December 6, 2019. doi:10.20452/pamw.15093
21. Bang L, Shivakumar M, Garg T, Kim D. Genetic Analysis Reveals Rare Variants in T-Cell Response Gene MR1 Associated with Poor Overall Survival after Urothelial Cancer Diagnosis. *Cancers*. 2021;13(8):1864. doi:10.3390/cancers13081864
22. Barrdahl M, Canzian F, Lindström S, et al. Association of breast cancer risk *loci* with breast cancer survival: Association of BC Risk *loci* with BC Survival. *Int J Cancer*. 2015;137(12):2837-2845. doi:10.1002/ijc.29446

23. Bayraktar S, Thompson PA, Yoo S, et al. The Relationship Between Eight GWAS-Identified Single- Nucleotide Polymorphisms and Primary Breast Cancer Outcomes. *The Oncologist*. 2013;18(5):493-500. doi:10.1634/theoncologist.2012-0419
24. Bi H, Yin J, Zhou L, et al. Clinicopathological and prognostic impact of somatic mutations in Chinese patients with clear cell renal cell carcinoma. *Transl Androl Urol*. 2020;9(6):2751-2763. doi:10.21037/tau-20-1410
25. Brand CU, Ellwanger U, Stroebel W, et al. Prolonged survival of 2 years or longer for patients with disseminated melanoma. :9.
26. Braun R, Finney R, Yan C, et al. Discovery Analysis of TCGA Data Reveals Association between Germline Genotype and Survival in Ovarian Cancer Patients. Toland AE, ed. *PLoS ONE*. 2013;8(3):e55037. doi:10.1371/journal.pone.0055037
27. Bristow RE, Gossett DR, Shook DR, et al. Micropapillary Serous Ovarian Carcinoma: Surgical Management and Clinical Outcome. *Gynecologic Oncology*. 2002;86(2):163-170. doi:10.1006/gyno.2002.6736
28. Bulian P, Rossi D, Forconi F, et al. IGHV gene mutational status and 17p deletion are independent molecular predictors in a comprehensive clinical-biological prognostic model for overall survival prediction in chronic lymphocytic leukemia. *J Transl Med*. 2012;10(1):18. doi:10.1186/1479-5876-10-18
29. Butkiewicz D, Rusin M, Sikora B, Lach A, Chorąży M. An association between DNA repair gene polymorphisms and survival in patients with resected non-small cell lung cancer. *Mol Biol Rep*. 2011;38(8):5231-5241. doi:10.1007/s11033-010-0674-1
30. Byun S-S, Heo TS, Choi JM, et al. Deep learning based prediction of prognosis in nonmetastatic clear cell renal cell carcinoma. *Sci Rep*. 2021;11(1):1242. doi:10.1038/s41598-020-80262-9
31. Cao S, Wang C, Huang X, Dai J, Hu L, Liu Y, Chen J, Ma H, Jin G, Hu Z, Xu L, Shen H. Prognostic assessment of apoptotic gene polymorphisms in non-small cell lung cancer in Chinese. *J Biomed Res*. 2013 May;27(3):231-8. doi: 10.7555/JBR.27.20130014. Epub 2013 Apr 25. PMID: 23720679; PMCID: PMC3664730.
32. Castro E, Romero-Laorden N, del Pozo A, et al. PROREPAIR-B: A Prospective Cohort Study of the Impact of Germline DNA Repair Mutations on the Outcomes of Patients With Metastatic Castration-Resistant Prostate Cancer. *JCO*. 2019;37(6):490-503. doi:10.1200/JCO.18.00358
33. Catasus L, Gallardo A, Cuatrecasas M, Prat J. Concomitant PI3K–AKT and p53 alterations in endometrial carcinomas are associated with poor prognosis. *Mod Pathol*. 2009;22(4):522-529. doi:10.1038/modpathol.2009.5
34. Catovsky D, Fooks J, Richards S. Prognostic factors in chronic lymphocytic leukaemia: the importance of age, sex and response to treatment in survival.: A REPORT FROM THE

MRC CLL 1 TRIAL. *Br J Haematol*. 1989;72(2):141-149. doi:10.1111/j.1365-2141.1989.tb07674.x

35. Chan JK, Zhang M, Kaleb V, et al. Prognostic factors responsible for survival in sex cord stromal tumors of the ovary—A multivariate analysis. *Gynecologic Oncology*. 2005;96(1):204-209. doi:10.1016/j.ygyno.2004.09.019
36. Chang S-C, Lin J-K, Yang SH, Wang H-S, Li AF-Y, Chi C-W. Relationship between genetic alterations and prognosis in sporadic colorectal cancer. *Int J Cancer*. 2006;118(7):1721-1727. doi:10.1002/ijc.21563
37. Chang TW, Kuo YL. A model building exercise of mortality risk for Taiwanese women with breast cancer. *BMC Med Inform Decis Mak*. 2010;10(1):43. doi:10.1186/1472-6947-10-43
38. Chang X, Shang F, Jiang H, et al. Clinicopathological Features and Prognostic Value of KRAS/NRAS/BRAF Mutations in Colorectal Cancer Patients of Central China. *CURR MED SCI*. 2021;41(1):118-126. doi:10.1007/s11596-021-2326-1
39. Chatrath A, Kiran M, Kumar P, Ratan A, Dutta A. The Germline Variants rs61757955 and rs34988193 Are Predictive of Survival in Lower Grade Glioma Patients. *Mol Cancer Res*. 2019;17(5):1075-1086. doi:10.1158/1541-7786.MCR-18-0996
40. Chatrath A, Przanowska R, Kiran S, et al. The pan-cancer landscape of prognostic germline variants in 10,582 patients. *Genome Med*. 2020;12(1):15. doi:10.1186/s13073-020-0718-7
41. Chen WS, Aggarwal R, Zhang L, et al. Genomic Drivers of Poor Prognosis and Enzalutamide Resistance in Metastatic Castration-resistant Prostate Cancer. *European Urology*. 2019;76(5):562-571. doi:10.1016/j.eururo.2019.03.020
42. Chetrit A, Hirsh-Yechezkel G, Ben-David Y, Lubin F, Friedman E, Sadetzki S. Effect of *BRCA1/2* Mutations on Long-Term Survival of Patients With Invasive Ovarian Cancer: The National Israeli Study of Ovarian Cancer. *JCO*. 2008;26(1):20-25. doi:10.1200/JCO.2007.11.6905
43. Choi JY, Kim JG, Lee YJ, et al. Prognostic Impact of Polymorphisms in the CASPASE Genes on Survival of Patients with Colorectal Cancer. *Cancer Res Treat*. 2012;44(1):32-36. doi:10.4143/crt.2012.44.1.32
44. Coombes CE, Abrams ZB, Li S, Abruzzo LV, Coombes KR. Unsupervised machine learning and prognostic factors of survival in chronic lymphocytic leukemia. *Journal of the American Medical Informatics Association*. 2020;27(7):1019-1027. doi:10.1093/jamia/ocaa060
45. Cui P-F, Cong X-F, Gao F, et al. Prognostic factors for overall survival in prostate cancer patients with different site-specific visceral metastases: A study of 1358 patients. *WJCC*. 2020;8(1):54-67. doi:10.12998/wjcc.v8.i1.54

46. Dai J, Hu Z, Dong J, et al. Host immune gene polymorphisms were associated with the prognosis of non-small-cell lung cancer in Chinese. *Int J Cancer*. 2012;130(3):671-676. doi:10.1002/ijc.26067
47. Delahunt B, Bethwaite P, Nacey JN. Renal cell carcinoma in New Zealand: A national survival study. *Urology*. 1994;43(3):300-309. doi:10.1016/0090-4295(94)90070-1
48. Deng F, Mu J, Qu C, et al. A Novel Prognostic Model of Endometrial Carcinoma Based on Clinical Variables and Oncogenomic Gene Signature. *Front Mol Biosci*. 2021;7:587822. doi:10.3389/fmolb.2020.587822
49. Diniz RW, Guerra MR, Cintra JRD, Fayer VA, Teixeira MTB. Disease-free survival in patients with non-metastatic breast cancer. *Rev Assoc Med Bras*. 2016;62(5):407-413. doi:10.1590/1806-9282.62.05.407
50. Dolatkhah R, Somi MH, Asvadi Kermani I, et al. Association between proto-oncogene mutations and clinicopathologic characteristics and overall survival in colorectal cancer in East Azerbaijan, Iran. *OTT*. 2016;Volume 9:7385-7395. doi:10.2147/OTT.S116373
51. Dong J, Hu Z, Shu Y, et al. Potentially functional polymorphisms in DNA repair genes and non-small-cell lung cancer survival: A pathway-based analysis: DNA REPAIR SNPs AND NSCLC SURVIVAL. *Mol Carcinog*. 2012;51(7):546-552. doi:10.1002/mc.20819
52. Dorjgochoo T, Zheng Y, Gao Y-T, et al. No association between genetic variants in angiogenesis and inflammation pathway genes and breast cancer survival among Chinese women. *Cancer Epidemiology*. 2013;37(5):619-624. doi:10.1016/j.canep.2013.06.005
53. Du H, Liu L, Liu H, et al. Genetic variants of DOCK2, EPHB1 and VAV2 in the natural killer cell-related pathway are associated with non-small cell lung cancer survival. :26.
54. Durbec F, Martin L, Derancourt C, Grange F. Melanoma of the hand and foot: epidemiological, prognostic and genetic features. A systematic review: Melanoma of the hand and foot. *British Journal of Dermatology*. 2012;166(4):727-739. doi:10.1111/j.1365-2133.2011.10772.x
55. Enders F, Geisenberger C, Jungk C, et al. Prognostic factors and long-term survival in surgically treated brain metastases from non-small cell lung cancer. *Clinical Neurology and Neurosurgery*. 2016;142:72-80. doi:10.1016/j.clineuro.2016.01.011
56. Faehling M, Schwenk B, Kramberg S, et al. Oncogenic driver mutations, treatment, and EGFR-TKI resistance in a Caucasian population with non-small cell lung cancer: survival in clinical practice. *Oncotarget*. 2017;8(44):77897-77914. doi:10.18632/oncotarget.20857
57. Falkson CI, Falkson HC. Prognostic factors in metastatic malignant melanoma. An analysis of 236 patients treated on clinical research studies at the Department of Medical Oncology, University of Pretoria, South Africa from 1972-1992. *Oncology*. 1998;55(1):59-64. doi:10.1159/000011836

58. Faradmal J, Roshanaei G, Mafi M, Sadighi-Pashaki A, Karami M. Application of Censored Quantile Regression to Determine Overall Survival Related Factors in Breast Cancer. *J Res Health Sci*. 2016;16(1):36-40.
59. Fernández-de-Misa Cabrera R, González Delgado B, Gambra Michel LE, et al. Clinicopathological characteristics of cutaneous malignant melanoma in patients at a tertiary hospital in Macaronesia. Survival as a function of locoregional prognostic factors per the American Joint Committee on Cancer. *Int J Dermatol*. 2018;57(2):193-201. doi:10.1111/ijd.13848
60. Ferri M, Lorenzon L, Onelli MR, et al. Lymph node ratio is a stronger prognotic factor than microsatellite instability in colorectal cancer patients: Results from a 7 years follow-up study. *International Journal of Surgery*. 2013;11(9):1016-1021. doi:10.1016/j.ijsu.2013.05.031
61. Ficarra V, Prayer-Galetti T, Novella G, et al. Incidental Detection beyond Pathological Factors as Prognostic Predictor of Renal Cell Carcinoma. *European Urology*. 2003;43(6):663-669. doi:10.1016/S0302-2838(03)00142-8
62. Ficarra V, Righetti R, Pilloni S, et al. Prognostic Factors in Patients with Renal Cell Carcinoma: Retrospective Analysis of 675 Cases. *European Urology*. 2002;41(2):190-198. doi:10.1016/S0302-2838(01)00027-6
63. FitzGerald LM, Zhao S, Leonardson A, et al. Germline variants in IL4, MGMT and AKT1 are associated with prostate cancer-specific mortality: An analysis of 12,082 prostate cancer cases. *Prostate Cancer Prostatic Dis*. 2018;21(2):228-237. doi:10.1038/s41391-017-0029-2
64. Fleming ST, Rastogi A, Dmitrienko A, Johnson KD. A comprehensive prognostic index to predict survival based on multiple comorbidities: a focus on breast cancer. *Med Care*. 1999;37(6):601-614. doi:10.1097/00005650-199906000-00009
65. Frank I, Blute ML, Cheville JC, Lohse CM, Weaver AL, Zincke H. An outcome prediction model for patients with clear cell renal cell carcinoma treated with radical nephrectomy based on tumor stage, size, grade and necrosis: the SSIGN score. *J Urol*. 2002;168(6):2395-2400. doi:10.1097/01.ju.0000035885.91935.d5
66. Fu F, Guo W, Lin Y, et al. Subtype-specific associations between breast cancer risk polymorphisms and the survival of early-stage breast cancer. *J Transl Med*. 2018;16(1):270. doi:10.1186/s12967-018-1634-0
67. Gallagher DJ, Vijai J, Cronin AM, et al. Susceptibility Loci Associated with Prostate Cancer Progression and Mortality. *Clin Cancer Res*. 2010;16(10):2819-2832. doi:10.1158/1078-0432.CCR-10-0028
68. Garbe C, Orfanos CE. Epidemiology of Malignant Melanoma in Central Europe: Risk Factors and Prognostic Predictors. Results of the Central Malignant Melanoma Registry

of the German Dermatological Society. *Pigment Cell Research*. 2008;3:285-294. doi:10.1111/j.1600-0749.1990.tb00387.x

69. Ge W, Cai W, Bai R, et al. A novel 4-gene prognostic signature for hypermutated colorectal cancer. *CMAR*. 2019;Volume 11:1985-1996. doi:10.2147/CMAR.S190963
70. Ghazali AK, Musa KI, Naing NN, Mahmood Z. Prognostic Factors in Patients With Colorectal Cancer at Hospital Universiti Sains Malaysia. *Asian Journal of Surgery*. 2010;33(3):127-133. doi:10.1016/S1015-9584(10)60022-X
71. Gohari MR, Biglarian A, Bakhshi E, Pourhoseingholi MA. Use of an artificial neural network to determine prognostic factors in colorectal cancer patients. *Asian Pac J Cancer Prev*. 2011;12(6):1469-1472.
72. Goodgame B, Viswanathan A, Zoole J, et al. Risk of Recurrence of Resected Stage I Non-small Cell Lung Cancer in Elderly Patients as Compared with Younger Patients. *Journal of Thoracic Oncology*. 2009;4(11):1370-1374. doi:10.1097/JTO.0b013e3181b6bc1b
73. Goodwin JS, Samet JM, Hunt WC. Determinants of Survival in Older Cancer Patients. *JNCI Journal of the National Cancer Institute*. 1996;88(15):1031-1038. doi:10.1093/jnci/88.15.1031
74. Gössling GCL, Chedid MF, Pereira FS, et al. Outcomes and Prognostic Factors of Patients with Metastatic Colorectal Cancer Who Underwent Pulmonary Metastasectomy with Curative Intent: A Brazilian Experience. *The Oncol*. Published online May 5, 2021:onco.13802. doi:10.1002/onco.13802
75. Griffith OL, Spies NC, Anurag M, et al. The prognostic effects of somatic mutations in ER-positive breast cancer. *Nat Commun*. 2018;9(1):3476. doi:10.1038/s41467-018-05914-x
76. Grotenhuis AJ, Dudek AM, Verhaegh GW, et al. Prognostic Relevance of Urinary Bladder Cancer Susceptibility Loci. Black PC, ed. *PLoS ONE*. 2014;9(2):e89164. doi:10.1371/journal.pone.0089164
77. Grotenhuis AJ, Dudek AM, Verhaegh GW, et al. Independent Replication of Published Germline Polymorphisms Associated with Urinary Bladder Cancer Prognosis and Treatment Response. *BLC*. 2016;2(1):77-89. doi:10.3233/BLC-150027
78. Gu C, Chen J, Dang X, et al. Hippo Pathway Core Genes Based Prognostic Signature and Immune Infiltration Patterns in Lung Squamous Cell Carcinoma. *Front Oncol*. 2021;11:680918. doi:10.3389/fonc.2021.680918
79. Gu C, Shi X, Qiu W, et al. Comprehensive Analysis of the Prognostic Role and Mutational Characteristics of m6A-Related Genes in Lung Squamous Cell Carcinoma. *Front Cell Dev Biol*. 2021;9:661792. doi:10.3389/fcell.2021.661792
80. Guo X, Li D, Wu Y, et al. Genetic variants in genes of tricarboxylic acid cycle key enzymes are associated with prognosis of patients with non-small cell lung cancer. *Lung Cancer*. 2015;87(2):162-168. doi:10.1016/j.lungcan.2014.12.005

81. Guo Y, Shi D, Zhang J, et al. The Hemoglobin, Albumin, Lymphocyte, and Platelet (HALP) Score is a Novel Significant Prognostic Factor for Patients with Metastatic Prostate Cancer Undergoing Cytoreductive Radical Prostatectomy. *J Cancer*. 2019;10(1):81-91. doi:10.7150/jca.27210
82. Guo Y, Feng Y, Liu H, et al. *Potentially Functional Genetic Variants in the C-Myb Signaling Pathway Genes Are Associated with Survival of Non-Small Cell Lung Cancer Patients in the PLCO Cancer Screening Trial*. In Review; 2019. doi:10.21203/rs.2.11515/v1
83. Hakimi AA, Chen Y-B, Wren J, et al. Clinical and Pathologic Impact of Select Chromatin-modulating Tumor Suppressors in Clear Cell Renal Cell Carcinoma. *European Urology*. 2013;63(5):848-854. doi:10.1016/j.eururo.2012.09.005
84. Hakimi AA, Mano R, Ciriello G, et al. Impact of Recurrent Copy Number Alterations and Cancer Gene Mutations on the Predictive Accuracy of Prognostic Models in Clear Cell Renal Cell Carcinoma. *Journal of Urology*. 2014;192(1):24-29. doi:10.1016/j.juro.2014.01.088
85. Haybittle J, Houghton J, Baum M. Social class and weight as prognostic factors in early breast cancer. *Br J Cancer*. 1997;75(5):729-733. doi:10.1038/bjc.1997.129
86. He Y, Liu H, Chen Q, Shao Y, Luo S. Relationships between SNPs and prognosis of breast cancer and pathogenic mechanism. *Mol Genet Genomic Med*. 2019;7(9). doi:10.1002/mgg3.871
87. He Y, Theodoratou E, Li X, et al. Effects of common genetic variants associated with colorectal cancer risk on survival outcomes after diagnosis: A large population- based cohort study. *Int J Cancer*. 2019;145(9):2427-2432. doi:10.1002/ijc.32550
88. He Y, Timofeeva M, Li X, et al. A Comprehensive Study of the Effect on Colorectal Cancer Survival of Common Germline Genetic Variation Previously Linked with Cancer Prognosis. *Cancer Epidemiol Biomarkers Prev*. 2019;28(11):1944-1946. doi:10.1158/1055-9965.EPI-19-0596
89. Hein A, Rack B, Li L, et al. Genetic Breast Cancer Susceptibility Variants and Prognosis in the Prospectively Randomized SUCCESS A Study. *Geburtshilfe Frauenheilkd*. 2017;77(06):651-659. doi:10.1055/s-0042-113189
90. Hippisley-Cox J, Coupland C. Development and validation of risk prediction equations to estimate survival in patients with colorectal cancer: cohort study. *BMJ*. Published online June 15, 2017:j2497. doi:10.1136/bmj.j2497
91. Hiroshige T, Eguchi Y, Yoshizumi O, et al. Baseline prostate-specific antigen levels following treatment with abiraterone acetate as a prognostic factor in castration-resistant prostate cancer. *Oncol Lett*. Published online March 14, 2018. doi:10.3892/ol.2018.8263

92. Huang C-Y, Chen B-H, Chou W-C, Yang C-T, Chang JW-C. Factors associated with the prognosis and long-term survival of patients with metastatic lung adenocarcinoma: a retrospective analysis. *J Thorac Dis*. 2018;10(4):2070-2078. doi:10.21037/jtd.2018.03.143
93. Huang L, Hu X, Wang Y, et al. Survival and pretreatment prognostic factors for extensive-stage small cell lung cancer: A comprehensive analysis of 358 patients. *Thorac Cancer*. 2021;12(13):1943-1951. doi:10.1111/1759-7714.13977
94. Hyman DM, Long KC, Tanner EJ, et al. Outcomes of primary surgical cytoreduction in patients with BRCA-associated high-grade serous ovarian carcinoma. *Gynecologic Oncology*. 2012;126(2):224-228. doi:10.1016/j.ygyno.2012.05.001
95. Ibrahim NI, Dahlui M, Aina EN, Al-Sadat N. Who are the Breast Cancer Survivors in Malaysia? *Asian Pacific Journal of Cancer Prevention*. 2012;13(5):2213-2218. doi:10.7314/APJCP.2012.13.5.2213
96. Itzykson R, Kosmider O, Renneville A, et al. Prognostic Score Including Gene Mutations in Chronic Myelomonocytic Leukemia. *JCO*. 2013;31(19):2428-2436. doi:10.1200/JCO.2012.47.3314
97. Jacot W, Quantin X, Boher J-M, et al. Brain metastases at the time of presentation of non-small cell lung cancer: a multi-centric AERIO* analysis of prognostic factors. *Br J Cancer*. 2001;84(7):903-909. doi:10.1054/bjoc.2000.1706
98. Jeske YW, Ali S, Byron SA, et al. FGFR2 mutations are associated with poor outcomes in endometrioid endometrial cancer: An NRG Oncology/Gynecologic Oncology Group study. *Gynecologic Oncology*. 2017;145(2):366-373. doi:10.1016/j.ygyno.2017.02.031
99. Ji G, Yao Q, Bao L, et al. Germline and tumor BRCA1/2 mutations in Chinese high grade serous ovarian cancer patients. *Ann Transl Med*. 2021;9(6):453-453. doi:10.21037/atm-20-6827
100. Jiang N, Xu X. Exploring the survival prognosis of lung adenocarcinoma based on the cancer genome atlas database using artificial neural network. *Medicine*. 2019;98(20):e15642. doi:10.1097/MD.0000000000015642
101. Jones CC, Bush WS, Crawford DC, et al. Germline Genetic Variants and Lung Cancer Survival in African Americans. *Cancer Epidemiol Biomarkers Prev*. 2017;26(8):1288-1295. doi:10.1158/1055-9965.EPI-16-0998
102. Kalady MF, White RR, Johnson JL, Tyler DS, Seigler HF. Thin Melanomas: Predictive Lethal Characteristics From a 30-Year Clinical Experience. *Annals of Surgery*. 2003;238(4):528-537. doi:10.1097/01.sla.0000090446.63327.40
103. Kang BW, Jeon H-S, Chae YS, et al. Association between GWAS-Identified Genetic Variations and Disease Prognosis for Patients with Colorectal Cancer. Cheah PY, ed. *PLoS ONE*. 2015;10(3):e0119649. doi:10.1371/journal.pone.0119649

104. Kang H-G, Lee YH, Lee SY, et al. *Genetic Variants in Histone Modification Regions Are Associated With the Prognosis of Lung Adenocarcinoma*. In Review; 2021. doi:10.21203/rs.3.rs-523665/v1

105. Kawaguchi T, Matsumura A, Fukai S, et al. Japanese Ethnicity Compared with Caucasian Ethnicity and Never-Smoking Status Are Independent Favorable Prognostic Factors for Overall Survival in Non-small Cell Lung Cancer: A Collaborative Epidemiologic Study of the National Hospital Organization Study Group for Lung Cancer (NHSGLC) in Japan and a Southern California Regional Cancer Registry Databases. *Journal of Thoracic Oncology*. 2010;5(7):1001-1010. doi:10.1097/JTO.0b013e3181e2f607

106. Kelty E, Ward SV, Cadby G, et al. Familial and non-familial risk factors associated with colorectal cancer survival in young and middle-aged patients. *Int J Colorectal Dis*. 2019;34(10):1673-1680. doi:10.1007/s00384-019-03380-5

107. Kihara R, Nagata Y, Kiyoi H, et al. Comprehensive analysis of genetic alterations and their prognostic impacts in adult acute myeloid leukemia patients. *Leukemia*. 2014;28(8):1586-1595. doi:10.1038/leu.2014.55

108. Kim HC, Ji W, Lee JC, et al. Prognostic Factor and Clinical Outcome in Stage III Non-Small Cell Lung Cancer: A Study Based on Real-World Clinical Data in the Korean Population. *Cancer Res Treat*. Published online February 16, 2021. doi:10.4143/crt.2020.1350

109. Kim J-Y, Lee E, Park K, et al. Clinical implications of genomic profiles in metastatic breast cancer with a focus on TP53 and PIK3CA, the most frequently mutated genes. *Oncotarget*. 2017;8(17):27997-28007. doi:10.18632/oncotarget.15881

110. Kim M, Kang H-G, Lee SY, et al. Comprehensive analysis of DNA repair gene polymorphisms and survival in patients with early stage non-small-cell lung cancer. *Cancer Science*. 2010;101(11):2436-2442. doi:10.1111/j.1349-7006.2010.01699.x

111. Kim SP, Feinglass J, Bennett CL, et al. Merging Claims Databases with a Tumor Registry to Evaluate Variations in Cancer Mortality: Results from a Pilot Study of 698 Colorectal Cancer Patients Treated at One Hospital in the 1990s. *Cancer Investigation*. 2004;22(2):225-233. doi:10.1081/CNV-120030211

112. Kim SH, Park B, Hwang EC, et al. Retrospective Multicenter Long-Term Follow-up Analysis of Prognostic Risk Factors for Recurrence-Free, Metastasis-Free, Cancer-Specific, and Overall Survival After Curative Nephrectomy in Non-metastatic Renal Cell Carcinoma. *Front Oncol*. 2019;9:859. doi:10.3389/fonc.2019.00859

113. Koessler T, Azzato EM, Perkins B, et al. Common germline variation in mismatch repair genes and survival after a diagnosis of colorectal cancer. *Int J Cancer*. 2009;124(8):1887-1891. doi:10.1002/ijc.24120

114. Koike T, Tsuchiya R, Goya T, Sohara Y, Miyaoka E. Prognostic Factors in 3315 Completely Resected Cases of Clinical Stage I Non-small Cell Lung Cancer in Japan. *Journal of Thoracic Oncology*. 2007;2(5):408-413. doi:10.1097/01.JTO.0000268674.02744.f9

115. Konopke R, Kersting S, Distler M, et al. Prognostic factors and evaluation of a clinical score for predicting survival after resection of colorectal liver metastases. *Liver International*. 2009;29(1):89-102. doi:10.1111/j.1478-3231.2008.01845.x

116. Korobeinikova E, Ugenskiene R, Insodaite R, et al. Association of angiogenesis and inflammation-related gene functional polymorphisms with early-stage breast cancer prognosis. *Oncol Lett*. Published online April 7, 2020. doi:10.3892/ol.2020.11521

117. Korobeinikova E, Ugenskiene R, Insodaite R, Rudzianskas V, Gudaitiene J, Juozaityte E. The role of functional polymorphisms in oxidative stress-related genes on early-stage breast cancer survival. *Int J Biol Markers*. 2021;36(2):14-21. doi:10.1177/17246008211011177

118. Kreklau A, Nel I, Kasimir-Bauer S, Kimmig R, Frackenpohl AC, Aktas B. An Observational Study on Breast Cancer Survival and Lifestyle Related Risk Factors. *In Vivo*. 2021;35(2):1007-1015. doi:10.21873/invivo.12344

119. Labussière M, Boisselier B, Mokhtari K, et al. Combined analysis of TERT, EGFR, and IDH status defines distinct prognostic glioblastoma classes. *Neurology*. 2014;83(13):1200-1206. doi:10.1212/WNL.0000000000000814

120. Lam VK, Lu A-TT, Kouzminova N, Lin AY. Characteristics of Colorectal Cancer Survival in an Urban County Hospital. *J Gastrointest Canc*. 2013;44(1):68-72. doi:10.1007/s12029-012-9446-6

121. Lee C-H, Cheng S-C, Tung H-Y, Chang S-C, Ching C-Y, Wu S-F. The Risk Factors Affecting Survival in Colorectal Cancer in Taiwan. *Iran J Public Health*. 2018;47:12.

122. Lee EB, Jeon H-S, Yoo SS, et al. Polymorphisms in Apoptosis-Related Genes and Survival of Patients with Early-Stage Non-Small-Cell Lung Cancer. *Ann Surg Oncol*. 2010;17(10):2608-2618. doi:10.1245/s10434-010-1082-4

123. Lee SY, Choi JE, Jeon H-S, et al. A Panel of Genetic Polymorphism for the Prediction of Prognosis in Patients with Early Stage Non-Small Cell Lung Cancer after Surgical Resection. Franco R, ed. *PLoS ONE*. 2015;10(10):e0140216. doi:10.1371/journal.pone.0140216

124. Li G, Wang G, Guo Y, et al. Development of a novel prognostic score combining clinicopathologic variables, gene expression, and mutation profiles for lung adenocarcinoma. *World J Surg Onc*. 2020;18(1):249. doi:10.1186/s12957-020-02025-0

125. Li H, Wang Y, Liu H, et al. Genetic variants of PDGF signaling pathway genes predict cutaneous melanoma survival. *Oncotarget*. 2017;8(43):74595-74606. doi:10.18632/oncotarget.20245
126. Li SW, Qiu XG, Chen BS, et al. Prognostic factors influencing clinical outcomes of glioblastoma multiforme. *Chin Med J (Engl)*. 2009;122(11):1245-1249.
127. Li Y-K, Xu Q, Sun L-P, et al. Nucleotide excision repair pathway gene polymorphisms are associated with risk and prognosis of colorectal cancer. *WJG*. 2020;26(3):307-323. doi:10.3748/wjg.v26.i3.307
128. Li Y, Wang X, Vural S, Mishra NK, Cowan KH, Guda C. Exome Analysis Reveals Differentially Mutated Gene Signatures of Stage, Grade and Subtype in Breast Cancers. Galli A, ed. *PLoS ONE*. 2015;10(3):e0119383. doi:10.1371/journal.pone.0119383
129. Liang J, Lv X, Lu C, et al. Prognostic factors of patients with Gliomas – an analysis on 335 patients with Glioblastoma and other forms of Gliomas. *BMC Cancer*. 2020;20(1):35. doi:10.1186/s12885-019-6511-6
130. Lin DW, FitzGerald LM, Fu R, et al. Genetic Variants in the *LEPR* , *CRY1* , *RNASEL* , *IL4* , and *ARVCF* Genes Are Prognostic Markers of Prostate Cancer-Specific Mortality. *Cancer Epidemiol Biomarkers Prev*. 2011;20(9):1928-1936. doi:10.1158/1055-9965.EPI-11-0236
131. Lindholm C, Andersson R, Dufmats M, et al. Invasive cutaneous malignant melanoma in Sweden, 1990-1999: A prospective, population-based study of survival and prognostic factors. *Cancer*. 2004;101(9):2067-2078. doi:10.1002/cncr.20602
132. Liu F, Zhao J, Xie J, et al. Prognostic risk factors in patients with bone metastasis from colorectal cancer. *Tumor Biol*. 2016;37(12):16127-16134. doi:10.1007/s13277-016-5465-4
133. Liu L, Liu H, Luo S, et al. Novel genetic variants of *SYK* and *ITGA1* related lymphangiogenesis signaling pathway predict non-small cell lung cancer survival. *Am J Cancer Res*. 2020;10(8):2603-2616. Published 2020 Aug 1.
134. Liu S, Zhang G, Li C, et al. Prognostic factors and survival of patients with small cell lung cancer in a northeastern Chinese population: Prognostic factors and survival in SCLC. *Thoracic Cancer*. 2013;4(2):143-152. doi:10.1111/j.1759-7714.2012.00149.x
135. Liu Y, Yasukawa M, Chen K, et al. Association of Somatic Mutations of *ADAMTS* Genes With Chemotherapy Sensitivity and Survival in High-Grade Serous Ovarian Carcinoma. *JAMA Oncol*. 2015;1(4):486. doi:10.1001/jamaoncol.2015.1432
136. Loi S, Michiels S, Lambrechts D, et al. Somatic Mutation Profiling and Associations With Prognosis and Trastuzumab Benefit in Early Breast Cancer. *JNCI: Journal of the National Cancer Institute*. 2013;105(13):960-967. doi:10.1093/jnci/djt121

137. Lu C-H, Chen I-H, Chen Y-J, et al. Primary Treatment and Prognostic Factors of Carcinosarcoma of the Ovary, Fallopian Tube, and Peritoneum: A Taiwanese Gynecologic Oncology Group Study. *Int J Gynecol Cancer*. 2014;24(3):506-512. doi:10.1097/IGC.0000000000000083

138. Lu G, Zhou B, He Y, et al. Novel genetic variants of *PIP5K1C* and *MVB12B* of the endosome-related pathway predict cutaneous melanoma-specific survival. *Am J Cancer Res*. 2020;10(10):3382-3394. Published 2020 Oct 1.

139. Ma X, Zhang Z, Chen X, et al. Prognostic factor analysis of patients with small cell lung cancer: Real- world data from 988 patients. *Thorac Cancer*. 2021;12(12):1841-1850. doi:10.1111/1759-7714.13846

140. Mahdy NH, Abdel-Fattah M, Ghanem H. Ovarian cancer in Alexandria from 1988 to 1997: trends and survival. East Mediterr Health J. 1999;5(4):727-739

141. Mahesh PA, Archana S, Jayaraj BS, et al. Factors affecting 30-month survival in lung cancer patients. *Indian J Med Res*. 2012;136(4):614-621.

142. Marcus EA, Weber TK, Rodriguez-Bigas MA, Driscoll D, Meropol NJ, Petrelli NJ. Prognostic Factors Affecting Survival in Patients with Colorectal Carcinomatosis. *Cancer Investigation*. 1999;17(4):249-252. doi:10.3109/07357909909040593

143. Marshall EM, Bertaut A, Desmoulins I, et al. Prognostic Factors of Survival among Women with Metastatic Breast Cancer and Impact of Primary or Secondary Nature of Disease on Survival: A French Population-Based Study. *Breast J*. 2017;23(2):138-145. doi:10.1111/tbj.12717

144. Masuda H, Kurita Y, Suzuki A, Kanbayashi T, Suzuki K, Fujita K. Prognostic Factors for Renal Cell Carcinoma: A Multivariate Analysis of 320 Cases. *Int J Urol*. 1997;4(3):247-253. doi:10.1111/j.1442-2042.1997.tb00181.x

145. McConechy MK, Talhouk A, Leung S, et al. Endometrial Carcinomas with *POLE* Exonuclease Domain Mutations Have a Favorable Prognosis. *Clin Cancer Res*. 2016;22(12):2865-2873. doi:10.1158/1078-0432.CCR-15-2233

146. Menor M, Zhu Y, Wang Y, Zhang J, Jiang B, Deng Y. Development of somatic mutation signatures for risk stratification and prognosis in lung and colorectal adenocarcinomas. *BMC Med Genomics*. 2019;12(S1):24. doi:10.1186/s12920-018-0454-7

147. Merriel SWD, Ingle SM, May MT, Martin RM. Retrospective cohort study evaluating clinical, biochemical and pharmacological prognostic factors for prostate cancer progression using primary care data. *BMJ Open*. 2021;11(2):e044420. doi:10.1136/bmjopen-2020-044420

148. Miles FL, Rao JY, Eckhert C, Chang SC, Pantuck A, Zhang ZF. Associations of immunity-related single nucleotide polymorphisms with overall survival among prostate cancer patients. *Int J Clin Exp Med*. 2015;8(7):11470-11476. Published 2015 Jun 24.

149. Mima K, Miyanari N, Morito A, et al. Frailty is an independent risk factor for recurrence and mortality following curative resection of stage I–III colorectal cancer. *Ann Gastroenterol Surg*. 2020;4(4):405-412. doi:10.1002/ags3.12337
150. Muranen TA, Khan S, Fagerholm R, et al. Association of germline variation with the survival of women with *BRCA1/2* pathogenic variants and breast cancer. *NPJ Breast Cancer*. 2020;6:44. Published 2020 Sep 10. doi:10.1038/s41523-020-00185-6
151. Nash GM, Gimbel M, Cohen AM, et al. KRAS Mutation and Microsatellite Instability: Two Genetic Markers of Early Tumor Development That Influence the Prognosis of Colorectal Cancer. *Ann Surg Oncol*. 2010;17(2):416-424. doi:10.1245/s10434-009-0713-0
152. Niu Y, Lin A, Luo P, et al. Prognosis of Lung Adenocarcinoma Patients With NTRK3 Mutations to Immune Checkpoint Inhibitors. *Front Pharmacol*. 2020;11:1213. doi:10.3389/fphar.2020.01213
153. O'Malley CD, Shema SJ, Cress RD, et al. The Implications of Age and Comorbidity on Survival Following Epithelial Ovarian Cancer: Summary and Results from a Centers for Disease Control and Prevention Study. *Journal of Women's Health*. 2012;21(9):887-894. doi:10.1089/jwh.2012.3781
154. Onida F, Kantarjian HM, Smith TL, et al. Prognostic factors and scoring systems in chronic myelomonocytic leukemia: a retrospective analysis of 213 patients. *Blood*. 2002;99(3):840-849. doi:10.1182/blood.V99.3.840
155. Ose J, Botma A, Balavarca Y, et al. Pathway analysis of genetic variants in folate-mediated one-carbon metabolism-related genes and survival in a prospectively followed cohort of colorectal cancer patients. *Cancer Med*. 2018;7(7):2797-2807. doi:10.1002/cam4.1407
156. Osmani F, Hajizadeh E, Akbari M. Prognostic Factors Associated with Curing in Patients with Breast Cancer: A Joint Frailty Model. *Int J Prev Med*. 2021;12(1):9. doi:10.4103/ijpvm.IJPVM_89_19
157. Ovcaricek T, Frkovic S, Matos E, Mozina B, Borstnar S. Triple negative breast cancer - prognostic factors and survival. *Radiology and Oncology*. 2011;45(1). doi:10.2478/v10019-010-0054-4
158. Padron E, Garcia-Manero G, Patnaik MM, et al. An international data set for CMML validates prognostic scoring systems and demonstrates a need for novel prognostication strategies. *Blood Cancer Journal*. 2015;5(7):e333-e333. doi:10.1038/bcj.2015.53
159. Parsons DW, Jones S, Zhang X, et al. An Integrated Genomic Analysis of Human Glioblastoma Multiforme. *Science*. 2008;321(5897):1807-1812. doi:10.1126/science.1164382

160. Patil R, Pandit P, Palwe V, et al. Evaluation of Prognostic Factors that Affect Survival Outcomes of Breast Cancer Patients with Brain Metastases: A Single Institutional Experience. *EJBH*. 2021;17(1):62-67. doi:10.4274/ejbh.2020.5969
161. Pereira B, Chin S-F, Rueda OM, et al. The somatic mutation profiles of 2,433 breast cancers refine their genomic and transcriptomic landscapes. *Nat Commun*. 2016;7(1):11479. doi:10.1038/ncomms11479
162. Pérez-Ramírez C, Cañadas-Garre M, Alnatsha A, et al. Interleukins as new prognostic genetic biomarkers in non-small cell lung cancer. *Surgical Oncology*. 2017;26(3):278-285. doi:10.1016/j.suronc.2017.05.004
163. Phipps AI, Passarelli MN, Chan AT, et al. Common genetic variation and survival after colorectal cancer diagnosis: a genome-wide analysis. *CARCIN*. 2016;37(1):87-95. doi:10.1093/carcin/bgv161
164. Piozzi GN, Park H, Lee TH, et al. Risk factors for local recurrence and long term survival after minimally invasive intersphincteric resection for very low rectal cancer: Multivariate analysis in 161 patients. *European Journal of Surgical Oncology*. Published online March 2021:S0748798321003917. doi:10.1016/j.ejso.2021.03.246
165. Pirie A, Guo Q, Kraft P, et al. Common germline polymorphisms associated with breast cancer-specific survival. *Breast Cancer Res*. 2015;17(1):58. Published 2015 Apr 22. doi:10.1186/s13058-015-0570-7
166. Poole EM, Konstantinopoulos PA, Terry KL. Prognostic implications of reproductive and lifestyle factors in ovarian cancer. *Gynecologic Oncology*. 2016;142(3):574-587. doi:10.1016/j.ygyno.2016.05.014
167. Putila J, Guo NL. Combining COPD with Clinical, Pathological and Demographic Information Refines Prognosis and Treatment Response Prediction of Non-Small Cell Lung Cancer. Minna JD, ed. *PLoS ONE*. 2014;9(6):e100994. doi:10.1371/journal.pone.0100994
168. Qian D, Liu H, Zhao L, et al. Novel genetic variants in genes of the Fc gamma receptor-mediated phagocytosis pathway predict non-small cell lung cancer survival. *Transl Lung Cancer Res*. 2020;9(3):575-586. doi:10.21037/tlcr-19-318
169. Rafiq S, Tapper W, Collins A, et al. Identification of Inherited Genetic Variations Influencing Prognosis in Early-Onset Breast Cancer. *Cancer Res*. 2013;73(6):1883-1891. doi:10.1158/0008-5472.CAN-12-3377
170. Rasouli MA, Moradi G, Roshani D, Nikkhoo B, Ghaderi E, Ghaytasi B. Prognostic factors and survival of colorectal cancer in Kurdistan province, Iran: A population-based study (2009–2014). *Medicine*. 2017;96(6):e5941. doi:10.1097/MD.0000000000005941

171. Ratto C, Sofo L, Ippoliti M, Merico M, Doglietto GB, Crucitti F. Prognostic factors in colorectal cancer. Literature review for clinical application. *Dis Colon Rectum*. 1998;41(8):1033-1049. doi:10.1007/BF02237397

172. Ribeiro HS de C, Stevanato-Filho PR, Costa Jr. WL da, Diniz AL, Herman P, Coimbra FJF. Prognostic factors for survival in patients with colorectal liver metastases: experience of a single brazilian cancer center. *Arq Gastroenterol*. 2012;49(4):266-272. doi:10.1590/S0004-28032012000400007

173. Ruden E, Reardon DA, Coan AD, et al. Exercise Behavior, Functional Capacity, and Survival in Adults With Malignant Recurrent Glioma. *JCO*. 2011;29(21):2918-2923. doi:10.1200/JCO.2011.34.9852

174. Safari M, Mahjub H, Esmaeili H, Abbasi M, Roshanaei G. Determining the Risk Factors Affecting on Death Due to Colorectal Cancer Progression: Survival Analysis in the Presence of Competing Risks. *J Gastrointest Canc*. Published online March 3, 2021. doi:10.1007/s12029-021-00609-x

175. Samorodnitsky S, Hoadley KA, Lock EF. A Pan-Cancer and Polygenic Bayesian Hierarchical Model for the Effect of Somatic Mutations on Survival. *Cancer Inform*. 2020;19:117693512090739. doi:10.1177/1176935120907399

176. Sanchez-Forgach ER, Mamounas EP, Warneke J, Driscoll D, Blumenson LE, Tsangaris TN. Factors affecting outcome in locally advanced breast cancer. *Surgical Oncology*. 1992;1(5):347-355. doi:10.1016/0960-7404(92)90035-J

177. Santos C, López-Doriga A, Navarro M, et al. Clinicopathological risk factors of Stage II colon cancer: results of a prospective study: Clinicopathological risk factors of colon cancer. *Colorectal Dis*. 2013;15(4):414-422. doi:10.1111/codi.12028

178. Scherer D, Deutelmoser H, Balavarca Y, et al. Polymorphisms in the Angiogenesis-Related Genes EFNB2, MMP2 and JAG1 Are Associated with Survival of Colorectal Cancer Patients. *IJMS*. 2020;21(15):5395. doi:10.3390/ijms21155395

179. Schildkraut J. Prognostic factors in early-onset epithelial ovarian cancer: a population-based study. *Obstetrics & Gynecology*. 2000;95(1):119-127. doi:10.1016/S0029-7844(99)00535-9

180. Schlenk RF, Döhner K, Krauter J, et al. Mutations and Treatment Outcome in Cytogenetically Normal Acute Myeloid Leukemia. *N Engl J Med*. 2008;358(18):1909-1918. doi:10.1056/NEJMoa074306

181. Seto Y, Kaneko Y, Mouri T, et al. Prognostic factors in older patients with wild-type epidermal growth factor receptor advanced non-small cell lung cancer: a multicenter retrospective study. *Transl Lung Cancer Res*. 2021;10(1):193-201. doi:10.21037/tlcr-20-894

182. Shen Y, Zhu Y-M, Fan X, et al. Gene mutation patterns and their prognostic impact in a cohort of 1185 patients with acute myeloid leukemia. *Blood*. 2011;118(20):5593-5603. doi:10.1182/blood-2011-03-343988
183. Shen Y, Han X, Wang J, et al. Prognostic impact of mutation profiling in patients with stage II and III colon cancer. *Sci Rep*. 2016;6(1):24310. doi:10.1038/srep24310
184. Sheridan J, Walsh P, Kevans D, et al. Determinants of short- and long-term survival from colorectal cancer in very elderly patients. *Journal of Geriatric Oncology*. 2014;5(4):376-383. doi:10.1016/j.jgo.2014.04.005
185. Shimamoto Y, Suga K, Nishimura J, Nawata H, Yamaguchi M. Major prognostic factors of Japanese patients with lymphoma-type adult T-cell leukemia. *Am J Hematol*. 1990;35(4):232-237. doi:10.1002/ajh.2830350403
186. Shin KM, Hong MJ, Lee SY, et al. Regulatory variants in cancer-related pathway genes predict survival of patients with surgically resected non-small cell lung cancer. *Gene*. 2018;646:56-63. doi:10.1016/j.gene.2017.12.055
187. Shu XO, Long J, Lu W, et al. Novel Genetic Markers of Breast Cancer Survival Identified by a Genome-Wide Association Study. *Cancer Res*. 2012;72(5):1182-1189. doi:10.1158/0008-5472.CAN-11-2561
188. Singal G, Miller PG, Agarwala V, et al. Association of Patient Characteristics and Tumor Genomics With Clinical Outcomes Among Patients With Non–Small Cell Lung Cancer Using a Clinicogenomic Database. *JAMA*. 2019;321(14):1391. doi:10.1001/jama.2019.3241
189. Song H, Hogdall E, Ramus SJ, et al. Effects of Common Germ-Line Genetic Variation in Cell Cycle Genes on Ovarian Cancer Survival. *Clin Cancer Res*. 2008;14(4):1090-1095. doi:10.1158/1078-0432.CCR-07-1195
190. Song N, Kim K, Shin A, et al. Colorectal cancer susceptibility loci and influence on survival. *Genes Chromosomes Cancer*. 2018;57(12):630-637. doi:10.1002/gcc.22674
191. Song Q, Seigne JD, Schned AR, Kelsey KT, Karagas MR, Hassanpour S. *A Machine Learning Approach for Long-Term Prognosis of Bladder Cancer Based on Clinical and Molecular Features*. Bioinformatics; 2019. doi:10.1101/557470
192. Song T, Seong SJ, Bae D-S, et al. Prognostic Factors in Women With Synchronous Endometrial and Ovarian Cancers. *Int J Gynecol Cancer*. 2014;24(3):520-527. doi:10.1097/IGC.0000000000000073
193. Steiner E, Eicher O, Sagemuller J, et al. Multivariate independent prognostic factors in endometrial carcinoma: A clinicopathologic study in 181 patients: 10 years experience at the Department of Obstetrics and Gynecology of the Mainz University. *Int J Gynecol Cancer*. 2003;13(2):197-203. doi:10.1046/j.1525-1438.2003.13021.x

194. Stevenson JD, McNair M, Cribb GL, Cool WP. Prognostic factors for patients with skeletal metastases from carcinoma of the breast. *The Bone & Joint Journal*. 2016;98-B(2):266-270. doi:10.1302/0301-620X.98B2.36185

195. Stremitzer S, Sunakawa Y, Zhang W, et al. Variations in genes involved in immune response checkpoints and association with outcomes in patients with resected colorectal liver metastases. *Pharmacogenomics J*. 2015;15(6):521-529. doi:10.1038/tpj.2015.14

196. Taieb J, Zaanan A, Le Malicot K, et al. Prognostic Effect of *BRAF* and *KRAS* Mutations in Patients With Stage III Colon Cancer Treated With Leucovorin, Fluorouracil, and Oxaliplatin With or Without Cetuximab: A Post Hoc Analysis of the PETACC-8 Trial. *JAMA Oncol*. 2016;2(5):643. doi:10.1001/jamaoncol.2015.5225

197. Takahashi R, Mabuchi S, Kawano M, et al. Prognostic significance of systemic neutrophil and leukocyte alterations in surgically treated endometrial cancer patients: A monoinstitutional study. *Gynecologic Oncology*. 2015;137(1):112-118. doi:10.1016/j.ygyno.2015.02.006

198. Tamura M, Matsumoto I, Tanaka Y, et al. Prognostic factor and treatment strategy for clinical N1 non-small cell lung cancer. *Gen Thorac Cardiovasc Surg*. 2020;68(3):261-265. doi:10.1007/s11748-019-01205-4

199. Tang D, Liu H, Zhao Y, et al. Genetic variants of *BIRC3* and *NRG1* in the NLRP3 inflammasome pathway are associated with non-small cell lung cancer survival. *Am J Cancer Res*. 2020;10(8):2582-2595. Published 2020 Aug 1.

200. Tantraworasin A, Saeteng S, Lertprasertsuke N, Arayawudhikule N, Kasemsarn C, Patumanond J. Completely Resected N0 Non-Small Cell Lung Cancer: Prognostic Factors Affecting Long-Term Survival. *ISRN Surgery*. 2013;2013:1-7. doi:10.1155/2013/175304

201. Tausch E, Beck P, Schlenk RF, Jebaraj BJ, Dolnik A, Yosifov DY, Hillmen P, Offner F, Janssens A, Babu GK, Grosicki S, Mayer J, Panagiotidis P, McKeown A, Gupta IV, Skorupa A, Pallaud C, Bullinger L, Mertens D, Döhner H, Stilgenbauer S. Prognostic and predictive role of gene mutations in chronic lymphocytic leukemia: results from the pivotal phase III study COMPLEMENT1. *Haematologica*. 2020 Oct 1;105(10):2440-2447. doi: 10.3324/haematol.2019.229161. PMID: 33054084; PMCID: PMC7556677.

202. Telepak LC, Jensen SE, Dodd SM, Morgan LS, Pereira DB. Psychosocial factors and mortality in women with early stage endometrial cancer. *Br J Health Psychol*. 2014;19(4):737-750. doi:10.1111/bjhp.12070

203. Theodoratou E, Farrington SM, Timofeeva M, et al. Genome-wide scan of the effect of common nsSNPs on colorectal cancer survival outcome. *Br J Cancer*. 2018;119(8):988-993. doi:10.1038/s41416-018-0117-7

204. Therkildsen C, Bergmann TK, Henrichsen-Schnack T, Ladelund S, Nilbert M. The predictive value of *KRAS, NRAS, BRAF, PIK3CA* and PTEN for anti-EGFR treatment in

metastatic colorectal cancer: A systematic review and meta-analysis. *Acta Oncologica*. 2014;53(7):852-864. doi:10.3109/0284186X.2014.895036

205. Tsuchiya N, Matsui S, Narita S, et al. Distinct Cancer-Specific Survival in Metastatic Prostate Cancer Patients Classified by a Panel of Single Nucleotide Polymorphisms of Cancer-Associated Genes. *Genes & Cancer*. 2013;4(1-2):54-60. doi:10.1177/1947601913481354

206. Vansteenkiste JF, De Leyn PR, Deneffe GJ, Lerut TE, Demedts MG. Clinical prognostic factors in surgically treated stage IIIA-N2 non-small cell lung cancer: analysis of the literature. *Lung Cancer*. 1998;19(1):3-13. doi:10.1016/S0169-5002(97)00072-X

207. Vidal GA, Carter GC, Gilligan AM, et al. Development of a Prognostic Factor Index Among Women With HR+/HER2− Metastatic Breast Cancer in a Community Oncology Setting. *Clinical Breast Cancer*. Published online January 2021:S1526820920303402. doi:10.1016/j.clbc.2020.12.012

208. Viganó A, Bruera E, Jhangri GS, Newman SC, Fields AL, Suarez-Almazor ME. Clinical Survival Predictors in Patients With Advanced Cancer. *Arch Intern Med*. 2000;160(6):861. doi:10.1001/archinte.160.6.861

209. Wang A, Holloway RW, Zhao Z, Zhang Z. *Effect of BRCA1 and BRCA2 Mutations on Endometrial Carcinoma Survival Rates*. In Review; 2020. doi:10.21203/rs.3.rs-24271/v1

210. Wang H, Liu H, Dai W, et al. Association of genetic variants of TMEM135 and PEX5 in the peroxisome pathway with cutaneous melanoma-specific survival. *Ann Transl Med*. 2021;9(5):396-396. doi:10.21037/atm-20-2117

211. Wang H, Yao Y, Li B. Factors associated with the survival of prostate cancer patients with rectal involvement. *Diagn Pathol*. 2014;9(1):35. doi:10.1186/1746-1596-9-35

212. Wang J, Hu G, Quan X. Analysis of the factors affecting the prognosis of glioma patients. *Open Medicine*. 2019;14(1):331-335. doi:10.1515/med-2019-0031

213. Wang L, Liu Y, Xu S. Prognostic factors for surgically managed patients with stage II non-small cell lung cancer. *Int J Clin Exp Med*. 2015;8(1):862-868. Published 2015 Jan 15.

214. Wang N, Lu Y, Khankari NK, et al. Evaluation of genetic variants in association with colorectal cancer risk and survival in Asians: GWAS identified SNPS and CRC risk and survival. *Int J Cancer*. 2017;141(6):1130-1139. doi:10.1002/ijc.30812

215. Wang R, Chen C, Jing Y, et al. Characteristics and prognostic significance of genetic mutations in acute myeloid leukemia based on a targeted next- generation sequencing technique. *Cancer Med*. 2020;9(22):8457-8467. doi:10.1002/cam4.3467

216. Wang X, Liu H, Xu Y, et al. Genetic variants in the calcium signaling pathway genes are associated with cutaneous melanoma-specific survival. *Carcinogenesis*. 2019;40(2):279-288. doi:10.1093/carcin/bgy188

217. Wang YA, Jian J-W, Hung C-F, et al. Germline breast cancer susceptibility gene mutations and breast cancer outcomes. *BMC Cancer*. 2018;18(1):315. doi:10.1186/s12885-018-4229-5
218. Watanabe T, Nanamiya H, Kojima M, et al. Clinical relevance of oncogenic driver mutations identified in endometrial carcinoma. *Translational Oncology*. 2021;14(3):101010. doi:10.1016/j.tranon.2021.101010
219. Wu Y, Liu Z, Tang D, et al. Potentially functional variants of HBEGF and ITPR3 in GnRH signaling pathway genes predict survival of non-small cell lung cancer patients. *Translational Research*. 2021;233:92-103. doi:10.1016/j.trsl.2020.12.009
220. Xie W, Stopsack KH, Drouin SJ, et al. Association of genetic variation of the six gene prognostic model for castration-resistant prostate cancer with survival. *Prostate*. 2019;79(1):73-80. doi:10.1002/pros.23712
221. Yang D, Khan S, Sun Y, et al. Association of BRCA1 and BRCA2 Mutations With Survival, Chemotherapy Sensitivity, and Gene Mutator Phenotype in Patients With Ovarian Cancer. *JAMA*. 2011;306(14):1557. doi:10.1001/jama.2011.1456
222. Yang L, Klint Å, Lambe M, et al. Predictors of ovarian cancer survival: A population-based prospective study in Sweden: Predictors of Ovarian Cancer Survival. *Int J Cancer*. 2008;123(3):672-679. doi:10.1002/ijc.23429
223. Yang S, Tang D, Zhao YC, et al. Potentially functional variants of ERAP1, PSMF1 and NCF2 in the MHC-I-related pathway predict non-small cell lung cancer survival. *Cancer Immunol Immunother*. Published online March 2, 2021. doi:10.1007/s00262-021-02877-9
224. Yazdani A, Yaseri M, Haghighat S, Kaviani A, Zeraati H. Investigation of Prognostic Factors of Survival in Breast Cancer Using a Frailty Model: A Multicenter Study. *Breast Cancer�(Auckl)*. 2019;13:117822341987911. doi:10.1177/1178223419879112
225. Yeole BB, Kumar AV, Kurkure A, Sunny L. Population-based survival from cancers of breast, cervix and ovary in women in Mumbai, India. *Asian Pac J Cancer Prev*. 2004;5(3):308-315.
226. Yin M, Grivas P, Wang Q, et al. Prognostic Value of DNA Damage Response Genomic Alterations in Relapsed/Advanced Urothelial Cancer. *The Oncol*. 2020;25(8):680-688. doi:10.1634/theoncologist.2019-0851
227. Yoo SS, Choi JE, Lee W-K, et al. Polymorphisms in the *CASPASE* Genes and Survival in Patients With Early-Stage Non–Small-Cell Lung Cancer. *JCO*. 2009;27(34):5823-5829. doi:10.1200/JCO.2009.23.1738
228. Yoo SS, Hong MJ, Jeon H-S, et al. Replication of results of a genome-wide association study on lung cancer survival in a Korean population. *Cancer Genetics*. 2014;207(1-2):35-39.e2. doi:10.1016/j.cancergen.2013.12.002

229. Yoon A, Park J-Y, Park J-Y, et al. Prognostic factors and outcomes in endometrial stromal sarcoma with the 2009 FIGO staging system: A multicenter review of 114 cases. *Gynecologic Oncology*. 2014;132(1):70-75. doi:10.1016/j.ygyno.2013.10.029

230. Yoon S, Kang BW, Park SY, et al. Prognostic relevance of genetic variants involved in immune checkpoints in patients with colorectal cancer. *J Cancer Res Clin Oncol*. 2016;142(8):1775-1780. doi:10.1007/s00432-016-2196-2

231. You W, Cai Z, Sheng N, et al. *Construction and Validation of a Clinicopathologic Signature for Predicting the Prognosis of Stage II and III Colorectal Cancer*. In Review; 2021. doi:10.21203/rs.3.rs-497202/v1

232. Zeng B, Ji P, Chen C, et al. A nomogram from the SEER database for predicting the prognosis of patients with non-small cell lung cancer. *The International Journal of Biochemistry & Cell Biology*. 2020;127:105825. doi:10.1016/j.biocel.2020.105825

233. Zhang C, Zheng Y, Li X, Hu X, Qi F, Luo J. Genome-wide mutation profiling and related risk signature for prognosis of papillary renal cell carcinoma. *Ann Transl Med*. 2019;7(18):427-427. doi:10.21037/atm.2019.08.113

234. Zhang H, Li Y, Guo S, et al. Effect of ERCC2 rs13181 and rs1799793 polymorphisms and environmental factors on the prognosis of patients with lung cancer. :13.

235. Zhang J, Wu Y, Hu X, et al. GSTT1, GSTP1, and GSTM1 genetic variants are associated with survival in previously untreated metastatic breast cancer. *Oncotarget*. 2017;8(62):105905-105914. doi:10.18632/oncotarget.22450

236. Zhang L, Gong Z. Clinical Characteristics and Prognostic Factors in Bone Metastases from Lung Cancer. *Med Sci Monit*. 2017;23:4087-4094. doi:10.12659/MSM.902971

237. Zhang M. Prognostic analysis and comparison of colon cancer in Han and Hui patients. *WJG*. 2014;20(17):5082. doi:10.3748/wjg.v20.i17.5082

238. Zhang M, Zhang N. Clinical and prognostic factors in 98 patients with malignant melanoma in China. *J Int Med Res*. 2017;45(4):1369-1377. doi:10.1177/0300060517708922

239. Zhao X, Little P, Hoyle AP, et al. The Prognostic Significance of Low-Frequency Somatic Mutations in Metastatic Cutaneous Melanoma. *Front Oncol*. 2019;8:584. doi:10.3389/fonc.2018.00584

240. Zhao Y, Ma R, Liu F, et al. *Analysis of Genome-Wide Mutation Profile and Establishment of Risk Signature for Prognosis of Bladder Cancer*. In Review; 2020. doi:10.21203/rs.3.rs-29487/v1

241. Zheng L, Zhan Y, Lu J, Hu J, Kong D. A prognostic predictive model constituted with gene mutations of APC, BRCA2, CDH1, SMO, and TSC2 in colorectal cancer. *Ann Transl Med*. 2021;9(8):680-680. doi:10.21037/atm-21-1010

242. Zheng P, Lai C, Yang W, Guo J, Xiao S, Chen Z. Nomogram predicting cancer-specific survival in elderly patients with stages I–III colon cancer. *Scandinavian Journal of Gastroenterology*. 2020;55(2):202-208. doi:10.1080/00365521.2020.1720280

243. Zheng X, Amos CI, Frost HR. Cancer prognosis prediction using somatic point mutation and copy number variation data: a comparison of gene-level and pathway-based models. *BMC Bioinformatics*. 2020;21(1):467. doi:10.1186/s12859-020-03791-0

244. Zhou B, Zhao YC, Liu H, et al. Novel Genetic Variants of ALG6 and GALNTL4 of the Glycosylation Pathway Predict Cutaneous Melanoma-Specific Survival. *Cancers*. 2020;12(2):288. doi:10.3390/cancers12020288

245. Zhou C, Gong X, Zhou D, Liang S. Analyses of prognostic factors in cases of non-small cell lung cancer with multiple brain metastases. *OTT*. Published online March 2016:977. doi:10.2147/OTT.S94524

246. Zhu H, Zhou L, Guo Y, et al. Factors for incidence risk and prognosis in non-small-cell lung cancer patients with synchronous brain metastasis: a population-based study. *Future Oncology*. 2021;17(19):2461-2473. doi:10.2217/fon-2021-0103

247. Zhu H, Dong D, Li F, et al. Clinicopathologic features and prognostic factors in patients with non-cutaneous malignant melanoma: a single-center retrospective study of 71 cases. *Int J Dermatol*. 2015;54(12):1390-1395. doi:10.1111/ijd.12745
